# Neuron-associated retroelement-derived protein Arc/Arg3.1 assists in the early stages of alphaherpesvirus infection in human neuronal cells

**DOI:** 10.1371/journal.pone.0314980

**Published:** 2024-12-12

**Authors:** Hiroko Kobayashi, Mitsuki Yasukochi, Masayuki Horie, Yasuko Orba, Hirofumi Sawa, Kan Fujino, Satoshi Taharaguchi

**Affiliations:** 1 Laboratory of Microbiology, School of Veterinary Medicine, Azabu University, Sagamihara, Kanagawa, Japan; 2 Division of Molecular Pathobiology, International Institute for Zoonosis Control, Hokkaido University, Sapporo, Hokkaido, Japan; 3 Laboratory of Veterinary Microbiology, Graduate School of Veterinary Science, Osaka Metropolitan University, Izumisano, Osaka, Japan; 4 Osaka International Research Center for Infectious Diseases, Osaka Metropolitan University, Osaka, Japan; 5 Institute for Vaccine Research and Development, Hokkaido University, Sapporo, Hokkaido, Japan; 6 One Health Research Center, Hokkaido University, Sapporo, Hokkaido, Japan; Arizona State University, UNITED STATES OF AMERICA

## Abstract

Alphaherpesviruses, including herpes simplex virus type 1 (HSV-1) and pseudorabies virus (PRV), are neurotropic double-stranded DNA viruses. Alphaherpesviruses control the expression of various host factors to ensure efficient infection and propagation. Recently, HSV-1 was found to upregulate Arc/Arg3.1 (Arc) expression, which is a retroelement-derived domesticated gene. Arc is associated with learning and neuroplasticity in host neuronal cells, and its abnormal expression leads to neurological disorders. However, the detailed mechanisms underlying the upregulation of Arc and its physiological significance in viral infections remain unclear. In this study, we found that PRV infection upregulated Arc expression *in vitro* and identified ICP0 and EP0, the transcriptional regulatory genes of HSV-1 and PRV, as triggers for enhanced Arc expression. Mass spectrometry and co-immunoprecipitation assays identified VP5, the major capsid protein of PRV and HSV-1, as the viral factor that interacted with Arc. *Arc* knockdown delayed viral infection during the early stages of the viral life cycle, but did not impact the viral attachment and entry. In conclusion, we provide evidence that alphaherpesvirus ICP0 homologues control Arc expression. Additionally, we demonstrate that Arc interacts with the major capsid protein VP5 and plays an important role in the viral lifecycle after intracellular entry. This study advances our knowledge of herpesvirus and retroelement-derived Arc interactions, providing fundamental insights into the pathogenesis of retroelement-derived domesticated genes and herpesvirus-induced neurological diseases.

## Introduction

Herpesviruses are double-stranded DNA viruses, genetically and biologically classified into three subfamilies: alpha-, beta-, and gamma-herpesviruses. Alphaherpesviruses, represented by herpes simplex virus type 1 (HSV-1) and pseudorabies virus (PRV), are known to be neurotropic and efficiently infect the peripheral nervous system after initial propagation in epithelial cells. After reaching the peripheral nervous cells, Alphaherpesviruses can migrate to the central nervous system through axonal transport mechanisms and induce severe neurological disorders [1–3].

During alphaherpesvirus infection, many host gene-expressions were controlled depending on the viral lifecycle. For example, HSV-1 infection could regulate host gene-expression through polymerase II and III [4, 5]. In the case of host genes *egr-1* and *c-fos*, which is expressed instantaneously, depends on neuronal activity and the protein expression level is altered by HSV-1 infection [6, 7].

Previous reports suggest that for *Arc/Arg3*.*1* (the activity-regulated cytoskeleton-associated protein, Arc), a similar type of host gene, HSV-1 infection enhances the expression of Arc mRNA and protein in the mouse brain *in vivo* and in human neural cultured cells *in vitro*, and *Arc* knockdown reduces the HSV-1 viral titer in human neural cultured cells *in vitro* [8]. Arc mRNA is mainly expressed in excitatory glutamatergic neurons of the central nervous system in mammals, and it was discovered in rat hippocampus by experimentally inducing electrical seizures *in vivo* [9]. In neuronal cells, Arc protein can traffic AMPA-type glutamate receptors via endocytic machinery, which depends on neural activity-driven signals. Hence, Arc protein is thought to play a role in the consolidation of enduring synaptic plasticity and memory storage [9–11]. Recent studies have also revealed that Arc originates from the Ty3/Gypsy retrotransposon gag element and is highly conserved in the genomes of several animals, from flies to humans [12–14]. Another study suggested that HSV-1 infection induced structural disassembly and functional deregulation in cultured cortical neurons, altered glutamate response, led to Arc protein accumulation within the somata, and decreased the expression of spine scaffolding-like proteins [15]. However, HSV-1 factors that induce Arc expression, and the mechanism of reduced HSV-1 titers owing to the lack of Arc remain unclear.

In this study, we used PRV and HSV-1 to analyze the mechanisms underlying Arc upregulation and its impact on the viral lifecycle of alphaherpesviruses, providing novel insights into the interactions between exogenous viruses and retroelement-associated host gene.

## Materials & methods

### Cells and viruses

Vero, HEK293T, and *Arc*-knockout HEK293T cells were cultured in Dulbecco’s modified Eagle’s medium (DMEM) (Thermo Fisher Scientific, Waltham, MA, USA), containing 10% FBS and an antibiotic mixture (penicillin, 20 IU/mL; streptomycin, 0.1 mg/mL). Human neuroblastoma SH-SY5Y cells were cultured on a collagen-coated plate in DMEM F12 (DMEM F12) (Thermo Fisher Scientific), containing 10% FBS and an antibiotic mixture (penicillin, 20 IU/mL; streptomycin, 0.1 mg/mL). SH-SY5Y cells were differentiated in Neurobasal Plus medium (Thermo Fisher Scientific), containing B27 supplement (Thermo Fisher Scientific), all-trans retinoic acid (Fujifilm, Wako, Japan), and GlutaMAX (Thermo Fisher Scientific). SH-SY5Y cells were differentiated, as previously described [16]. All cells were incubated at 37°C in the presence of 5% CO_2_. PRV YS-81 and HSV-1 F strains were propagated in Vero cells in DMEM, containing an antibiotic mixture (penicillin, 20 IU/mL; streptomycin, 0.1 mg/mL). After propagation, viruses were stored at -80°C. To inactivate the virus using UV light, the virus solution was placed 10 cm directly below the UV-C light source and exposed to a total fluence of 20 J/cm^2^ over 30 min [17, 18].

### Primers

The primers used to construct the plasmids and those for real-time qPCR and guide RNA (gRNA) sequences for producing *Arc*-knockout cells are listed in Table 1.

**Table 1:** Primer sequences for plasmid construction, gRNA construction, and qRT-PCR.

Purpose	Primer name	Sequence (5’→3’)
**Plasmid construction**	HA-EP0_F	CATGGTACCATGTACCCATACGATGTTCCAGATTACGCTATGGACTGCCCCATCTGTCTG
	EP0_R	ATGGAATTCTCAGTCGTCGTCCTGGGTGAG
	HA-UL54_F	CATGGTACCATGTACCCATACGATGTTCCAGATTACGCTATGGAGGACAGCGGCAACAG
	UL54_R	ATGGAATTCTCAAAACAGGTGGTTGCAGTAA
	HA-UL48_F	CATGGTACCATGTACCCATACGATGTTCCAGATTACGCTATGCGCGACGAGGAGTGCGTG
	UL48_R	ATGGAATTCTCACATCTCAAACATCCGGTTG
	IE180_F	CATGGTACCATGGCCGACGATCTCTTTGAC
	IE180_R	ATGGAATTCTCAGCGGAGCAGCAGGTAGGG
	ICP0_HA_F	CATGAATTCGACCATGTACCCATACGATGTTCCAGATTACGCTATGGAGCCCCGCCCCGGA
	ICP0_R	ATGGCGGCCGCTTATTGTTTTCCCTCGTCCCGG
	HSV-1-VP5_F	CATGGTACCATGGCCGCTCCCAACCGCGAC
	HSV-1-VP5_R	ATGGCGGCCGCTTATTTGTCATCGTCGTCCTTGTAGTCCAGAGCCAGTCCCTTGAGCG
	PRV-VP5_F	CATGGTACCATGGAGCGCCCGGCCATCCTGC
	PRV-VP5_R	ATGGAATTCTCATTTGTCATCGTCGTCCTTGTAGTCGGCGCTCGCGTGCTGGAAG
**gRNA construction**	Arc_430–449_F	CCGGGCCCGCCACACCGTTTCCGT
	Arc_430–449_R	AAACACGGAAACGGTGTGGCGGGC
**qRT-PCR**	hsArc_F	GGAGTACTGGCTGTCCCAGA
	hsArc_R	ACTCCACCCAGTTCTTCACG
	hsGAPDH_F	AAGGTCATCCCTGAGCTGAA
	hsGAPDH_R	TGCTGTAGCCAAATTCGTTG
	PRV_gB/UL27_F	ACTACGAGGACTACAACTACGTGCG
	PRV_gB/UL27_R	GTCACCCGCGTGCTGATC
	HSV-1-VP5_F	AACAGCCTGTACGACGTC
	HSV-1-VP5_R	AACTTGGTACACACGCACGC
	hsTP53_F	CCCTTCCCAGAAAACCTACC
	hsTP53_R	CAGGCATTGAAGTCTCATGG

### Plasmid construction

*Arc*, *EP0*, *UL48*, *UL54*, and *UL19* genes were amplified by PCR using cDNA templates derived from differentiated SH-SY5Y cells infected with PRV at an MOI of 1. ICP0 and VP5 were amplified by PCR using cDNA templates derived from differentiated SH-SY5Y cells infected with HSV-1 at an MOI of 1. All the PCR products were inserted into the pEF4A vector. The Arc, EP0, UL48, UL54, and ICP0 expression plasmids contained an HA-tag sequence inserted at the N-terminus for detection. The PRV or HSV-1 VP5 expression plasmids also contained a FLAG-tag. For the TAP assay, an Arc expression plasmid tagged with HA and FLAG was prepared. IE180 cloned into the pc/IE vector [19] was amplified by PCR and cloned into the pEF4A vector. Nucleotide sequences were confirmed by Sanger sequencing.

### Western blotting

Cells were lysed and boiled for 5 min at 98°C with SDS-PAGE loading buffer. Proteins from each sample were separated by SDS-PAGE and transferred onto hydrophilic membranes. The membranes were blocked with 5% skim milk in phosphate-buffered saline with 0.1% Tween 20 (PBST) for 1 h at 25°C, except when using anti-tubulin (proteintech, Rosemont, IL, USA), for which blocking was performed for 2 h. After blocking, the membranes were incubated for 1 h at 25°C with the primary antibodies: anti-HA (Abcam, Cambridge, MA, USA), anti-IE180 [20], anti-GAPDH (GeneTex, Irvine, CA, USA), anti-ACTB (GeneTex), anti-Arc (Santa Cruz, Dallas, TX, USA), anti-HSV-1 antibody (Agilnet, Santa Clara, CA, USA), anti-tubulin and anti-histone (Sigma-Aldrich, St. Louis, MO, USA). Each antibody was diluted 1,000-fold prior to use, except for anti-tubulin (20,000-fold) and anti-histone (10,000-fold). The membranes were washed thrice with PBST and incubated with anti-rabbit HRP antibody (Bio Rad, Hercules, CA, USA) or anti-mouse HRP antibody (Bio Rad) for 1 h at 25°C. Anti-rabbit and anti-mouse HRP antibodies were diluted 3000-fold before use. The signal intensity was determined using an Amersham ImageQuant 800. To quantify the amount of protein, the target band intensities were calculated using Image J. Western blot raw images are available in the supporting files (S1 Raw images).

### Quantitative real-time PCR

Total RNA was extracted from cells using TriPure Isolation Reagent (Sigma-Aldrich) or TRIzol reagent (Invitrogen, Waltham, MA, USA) and a Direct-zol RNA kit (Zymo Research, Irvine, CA, USA). cDNA was synthesized by reverse transcription of the total RNA extract using a Verso cDNA Synthesis Kit (Thermo Fisher Scientific). Total DNA was extracted from the cells using a DNeasy Blood & Tissue Kit (Qiagen, Hilden, Germany). The mRNA or DNA levels of the target genes were quantified using the TB Green Premix Ex Taq II kit (Takara, Shiga, Japan) or THUNDERBIRD SYBR qPCR Mix (TOYOBO, Osaka, Japan).

### *Arc* knockdown by siRNA

For *Arc* knockdown in SH-SY5Y cells, two custom siRNAs (Silencer™ Select Pre-Designed siRNA Assay ID s23361: si#1 and ID s23362: si#2) and Silencer Select Negative Control No. 1 siRNA (Ctrl) were used in this study. SH-SY5Y cells were seeded in a collagen-coated plate and transfected with 50 nM of each siRNA using Lipofectamine RNAiMAX (Invitrogen) twice, one day apart, during the differentiation process. Then, 24 h after the second siRNA treatment, the cells were used for infection experiments.

### Construction of *Arc*-knockout HEK293T cells

The gRNA sequence required for *Arc* knockout was determined using CHOPCHOP. Using the Guide-it™ CRISPR/Cas9 System (Red) (Takara), gRNA was cloned into the pGuide-it- tdTomato Vector plasmid. The gRNA expression plasmid was transfected into HEK293T cells, which were incubated for 48 h. Single cells with red fluorescence confirmed by fluorescence microscopy were cultured, and clones wherein Arc protein was not detected and a frameshift was confirmed on the Arc gene sequence were cultured. This cell clone was designated as *Arc*-knockout HEK293T and was used for TAP assay experiments.

### TAP assay

Arc-FLAG-HA-expressing plasmids were transfected into *Arc*-knockout HEK293T cells. After 24 h, PRV was inoculated into transfected *Arc*-knockout HEK293T cells at an MOI of 5. Infected cell lysates were collected at 24 hpi, and the proteins interacting with Arc were purified using a FLAG HA TAP Generation Kit (Sigma-Aldrich), following the manufacturer’s protocol.

### Mass spectrometry

TAP assay purified proteins were separated by SDS-PAGE and stained by a Silver Staining Kit for MS (Apro Science, Tokushima, Japan). Proteins purified from infected cells were identified using mass spectrometry at Japan Proteomics Co., Ltd.

### Co-IP assay

For the co-immunoprecipitation (co-IP) assay, *Arc*-knockout HEK293T cells were co-transfected with plasmids expressing HA-Arc and Flag-VP5. Then, 24 h after transfection, the cells were washed with PBS, lysed with NP-40 lysis buffer containing 150 mM NaCl, 20 mM Tris-HCl (pH 7.4), 1 mM EDTA and a protease inhibitor, and homogenized using a sonicator. The cell lysates were centrifuged at 18,800 × *g* for 20 min at 4°C. A portion of the supernatants was dissolved in the sample buffer and analyzed as an input sample. Protein G beads (TAMAGAWA SEIKI, Nagano, Japan) were first incubated with mouse anti-FLAG M2 antibody (Sigma-Aldrich) at 25°C for 30 min. The FLAG-antibody conjugated beads were collected and washed thrice with NP-40 lysis buffer. The cell lysis samples were mixed with the beads for 5 h at 4°C with rotating. Beads that reacted with the samples were collected and washed thrice with NP-40 lysis buffer. Proteins bound to the beads were eluted in sample buffer and analyzed by western blotting. Detection with each antibody was performed on parallel blots using the same lysate.

### Attachment and entry assay

For the attachment assay, siRNA-treated and differentiated SH-SY5Y cells were infected with PRV or HSV-1 at an MOI of 10 at 4°C for 1 h and washed with PBS followed by cellular DNA extraction. As a positive control, cells were also inoculated with each virus treated with heparin (final concentration: 50 units/mL) for 30 min at 37°C before use, following previous studies [21, 22]. For the entry assay, cells were infected with PRV or HSV-1 at an MOI of 10 at 37°C for 1 h and treated with trypsin for 5 min. To remove trypsin, the cells were centrifuged at 750 × *g* for 5 min and cellular DNA was extracted. The cell-attached and internalized virions after cell entry were analyzed by measuring the number of viral genome copies using real-time qPCR.

### Statistical analysis

All statistical analyses were performed using Microsoft Excel. The statistical significance of the data was determined using Student’s *t*-test, and *P* < 0.05 was considered as statistically significant. * indicates a statistically significant difference (* *P < *0.05; ** *P < 0*.*01*).

## Results

### Arc/Arg3.1 expression is enhanced by PRV infection

**Fig 1:**
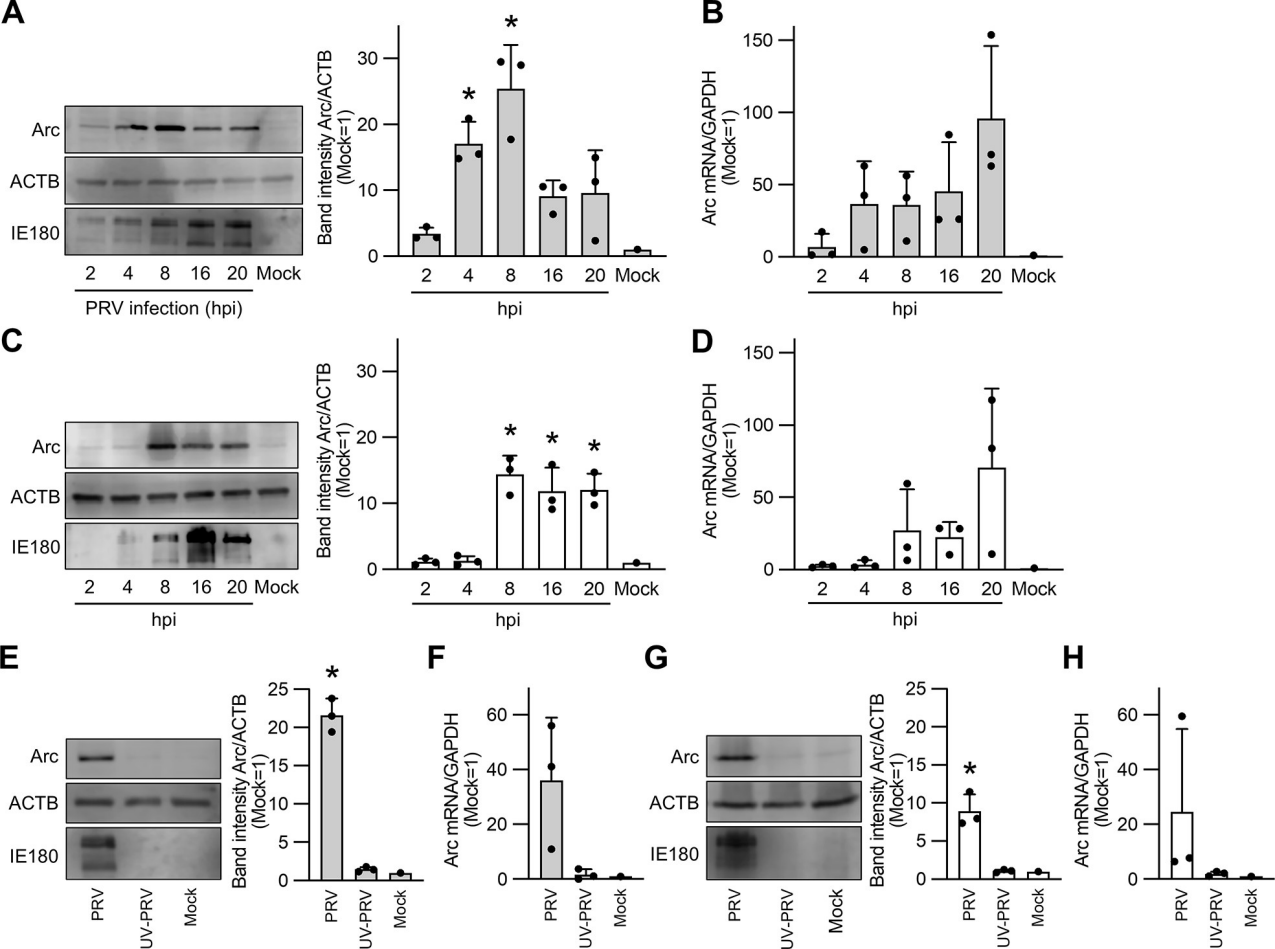
Arc/Arg3.1 expression is enhanced by PRV infection. Expression of Arc protein in neuron-differentiated SH-SY5Y cells (A, E) or HEK293T cells (C, G) and quantification of Arc mRNA in neuron-differentiated SH-SY5Y (B, F) and HEK293T cells (D, H) following PRV infection. Cells were infected with PRV at an MOI of 10, and cell lysates were collected for the detection of Arc protein by western blotting and Arc mRNA quantification by real-time RT-PCR. The cells in panels E, F, G, and H were inoculated with either PRV or UV-inactivated PRV. Samples were collected at 8 h post infection (hpi) (E, F, G, H) or at the indicated time points (A, B, C, D). Uninfected cells served as mock controls. PRV infection was validated by detecting the expression of PRV IE180 protein using western blotting. Relative protein levels were normalized to the amount of β-actin (ACTB). The band intensity was quantified using Image J software, with each band’s intensity compared to the mock set at 1. mRNA levels were normalized to the mean level of GAPDH mRNA, and the relative values were compared to those of the mock. Data are presented as the mean ± SEM from three independent experiments, with statistical significance determined by Student’s *t*-test (*P < 0.05).

The association between Arc expression and other alphaherpesviruses has not been characterized, except for HSV-1. In this study, we investigated whether PRV infection enhances Arc expression. Neuronally differentiated SH-SY5Y cells, derived from human neuroblastoma, and HEK293T cells were infected with PRV at an MOI of 10, and the expression level of Arc protein in the infected cells was detected by western blotting at 2–20 h post-infection (hpi) (Fig 1A and 1C). Differentiation of SH-SY5Y cells was confirmed morphologically (S1 Fig). The amount of Arc mRNA was quantified using real-time qPCR at the same time points (Fig 1B and 1D). The results showed that Arc protein expression was significantly upregulated in differentiated SH-SY5Y cells at 4 hpi, reaching the maximum expression levels at 8 hpi (Fig 1A). In HEK293T cells, a significant increase in Arc protein expression was observed at 8 hpi when compared to that in mock cells (Fig 1C). PRV infection also induced Arc mRNA expression, with maximum levels observed at 20 hpi in both cell types (Fig 1B and 1D). These results show that Arc expression is induced not only by HSV-1, but also by PRV infection, suggesting that this upregulation of Arc is common among alphaherpesvirus infections.

Next, to investigate whether viral replication is essential for Arc upregulation, we inoculated SH-SY5Y (Fig 1E and 1F) and HEK293T (Fig 1G and 1H) cells with PRV or UV-inactivated PRV and quantified the amount of Arc protein or mRNA at 8 hpi at an MOI of 10. The expression levels of the Arc protein (Fig 1E and 1G) and mRNA (Fig 1F and 1H) did not differ from those in the mock cells. A previous study reported that, when UV-treated HSV-1 and HSV-1 lacking each viral protein (US11, US3, and ICP0) infects the primary culture of human neurons, Arc protein was not expressed when compared with wild type-HSV-1 infection [8]. They revealed that HSV-1 replication was mandatory to induce Arc expression in HSV-1 infection. Although we cannot rule out the possibility that UV treatment in the present study affected not only the viral genome but also the viral proteins of PRV, thereby influencing stages such as particle attachment and entry, our data, in consideration of previous studies, suggest that PRV replication is essential for Arc upregulation.

### Arc/Arg3.1 expression is induced by alphaherpesvirus’ early protein

**Fig 2:**
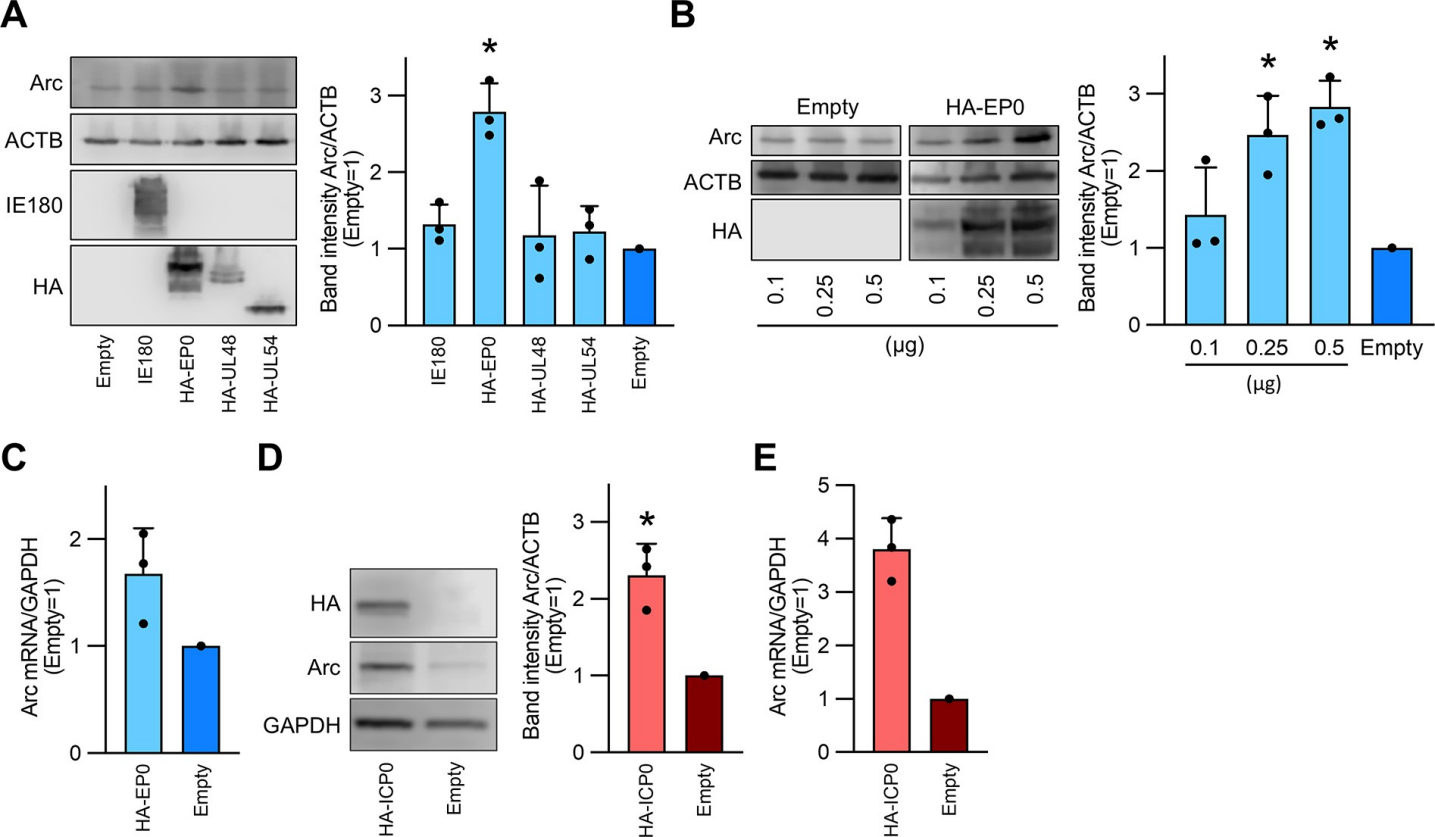
Arc/Arg3.1 expression is induced by alphaherpesvirus’ early protein. (A) HEK293T cells were transfected with 0.25 μg of PRV IE180, HA-tagged EP0 (HA-EP0), HA-tagged UL48 (HA-UL48), and HA-tagged UL54 (HA-UL54) expression plasmids. (B) Dose-dependent increase of Arc protein expression through HA-EP0 transfection. HEK293T cells were transfected with the indicated amounts of HA-EP0 (0.1, 0.25, and 0.5 μg). (D) Increase in Arc protein expression by HA-tagged ICP0. The expression level of Arc protein in each cell lysate at 24 h post-transfection was assessed by western blotting. Band intensities were quantified using Image-J software. The transfection efficacy was confirmed through the detection of each HA protein or IE180 protein, using their corresponding antibodies. Relative protein levels were normalized to the amount of ACTB or GAPDH. The intensity of each band was compared with the empty vector serving as the control (set as 1). (C and E) Arc mRNA levels were measured by real-time PCR at 24 h post-transfection when HEK293T cells were transfected with 0.25 μg of either HA-EP0 (C) or HA-ICP0 (E) expression plasmids. Relative Arc mRNA levels were normalized to the average of GAPDH mRNA levels. The results are presented as the mean ± SEM of three independent experiments (Student’s *t*-test, *P < 0.05).

As the induction of Arc protein expression began at 4 or 8 hpi and was dependent on the viral replication cycle, we hypothesized that viral transcriptional regulators elicited Arc expression. Therefore, we tested whether representative PRV transcriptional regulators, such as IE180, EP0, UL54, and UL46, could trigger Arc transcription. To confirm protein expression, a plasmid encoding each transcriptional regulator was constructed with an HA tag at the N-terminal domain. HEK293T cells were transfected with each plasmid, and the Arc protein was quantified by western blotting at 24 h post-transfection. The results showed that Arc protein expression significantly increased in EP0-transfected cells when compared to that in empty vector-transfected cells (Fig 2A). In addition, Arc expression was controlled in an EP0 dose-dependent manner (Fig 2B). Overexpression of ICP0, the HSV-1 homologue of EP0, caused a significant increase in Arc protein expression in transfected cells when compared to that in empty vector-transfected cells (Fig 2D). In transfected cells, Arc mRNA expression was also upregulated by EP0 and ICP0 (Fig 2C and 2E). These results indicate that Arc expression is activated by the viral early gene EP0 in PRV infection and by ICP0 in HSV-1 infection.

### *Arc* knockdown delays the early stages of alphaherpesvirus infection but does not affect viral attachment and entry

**Fig 3:**
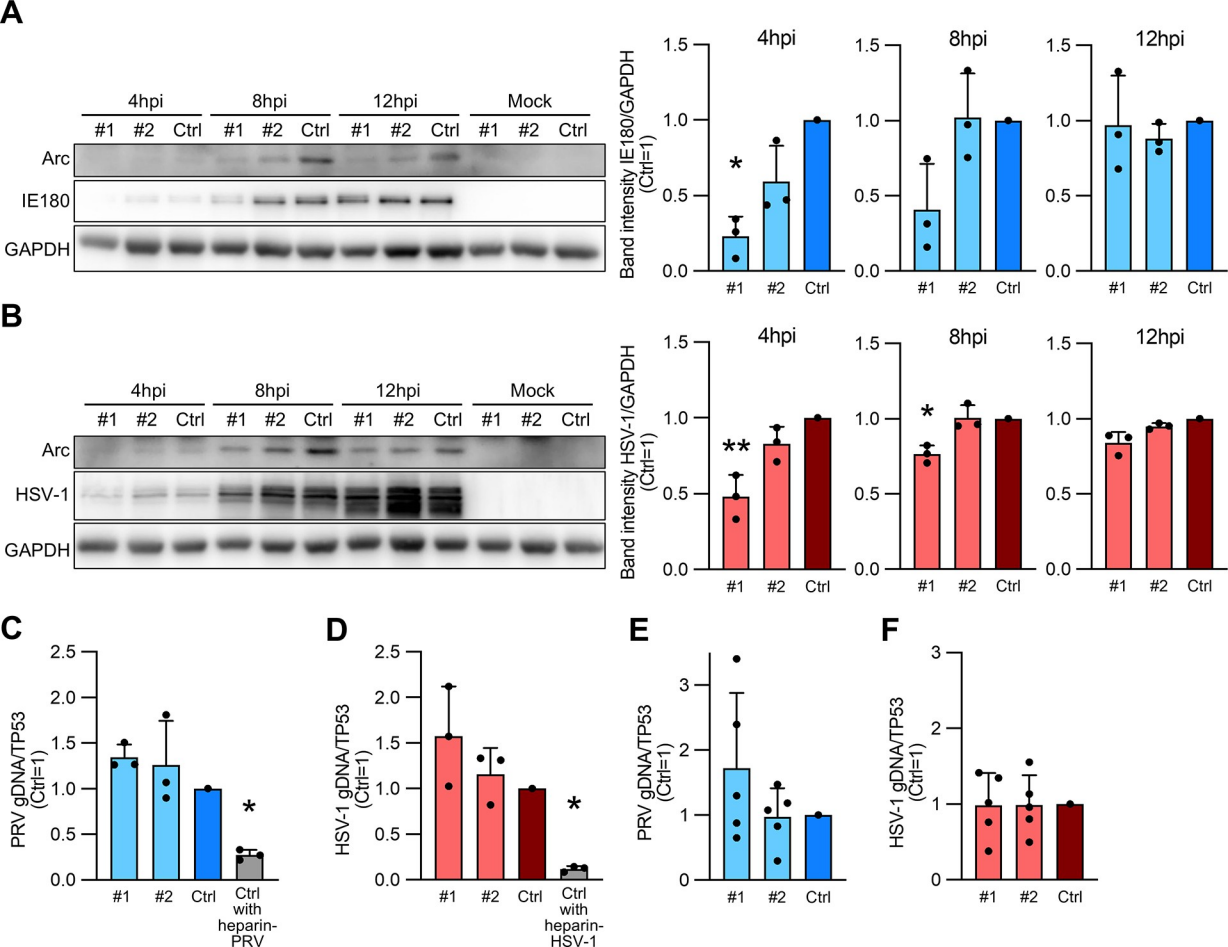
The initial infection of alphaherpesvirus is delayed by *Arc* knockdown. Neuronally differentiated SH-SY5Y cells were treated with siRNA #1 (#1) or siRNA #2 (#2) for *Arc* knockdown. Negative target siRNA-treated cells served as controls (Ctrl). PRV- (A) or HSV-1-infected (B) cells at an MOI of 10. The amount of PRV or HSV-1 was determined by western blotting for IE180 protein or the amount of protein detected by a polyclonal antibody against HSV-1. Relative protein levels were normalized to GAPDH protein levels. The band intensity was measured using ImageJ, and each band intensity was compared to that of the Ctrl (set as 1). (C, D) Attachment assay. (E, F) Entry assay. PRV (C, E) or HSV-1 (D, F) infected at an MOI of 10 with *Arc* knockdown neuron-differentiated SH-SY5Y cells for 1 h at 4°C for the attachment assay and 37°C for the entry assay. As a positive control in attachment assay, Ctrl cells treated with heparin-coated virus were prepared (Ctrl with heparin-PRV or Ctrl with heparin-HSV-1) (C, D). The expression levels of the intracellular viral gDNA were quantified by real-time PCR. The amounts of viral gDNA were normalized to the average of *TP53*, the cellular gene. The values are presented as the mean ± SEM of three or five independent experiments (Student’s *t*-test, *P < 0.05, **P < 0.01).

To test whether Arc expression has any effect on viral infection, we treated neuronally differentiated SH-SY5Y cells with *Arc* knockdown with two different siRNAs (#1 and #2), and *Arc* knockdown cells were infected with PRV or HSV-1 at an MOI of 10. Intracellular viral proteins were confirmed at 4, 8, and 12 hpi by western blotting. The results showed that the expression of PRV-IE180 was significantly reduced in knockdown cells when compared to that in the negative control siRNA (Ctrl) treated cells at 4 hpi (Fig 3A). Furthermore, IE180 protein expression tended to decrease even at 8 hpi in *Arc* knockdown cells (Fig 3A). The expression level of HSV-1 was significantly reduced at 8 hpi in knockdown cells when compared to that in Ctrl cells (Fig 3B). There were no differences at 12 hpi between PRV- and HSV-1- infected *Arc* knockdown cells and the control.

These results indicate that *Arc* knockdown contributes to the reduction of alphaherpesvirus infection in the early stages of the viral life cycle before 12 hpi, suggesting that the virus may induce Arc through transcriptional regulators to promote infection for its own sake.

It was reported that following the cellular entry, HSV-1 traverses the cytoplasm and reaches the nucleus, wherein viral genome replication takes place at 3–12 hpi. Moreover, the expression and translation of α and β genes occur by approximately 4 hpi [23]. In the present study, to verify the effect of Arc on the attachment and entry stages using methods adapted from previous studies on PRV and other viruses [24, 25], we infected *Arc* knockdown SH-SY5Y cells undergoing neuronal differentiation with PRV or HSV-1 at an MOI of 10, and intracellular viral genomic DNA was quantified by real-time qPCR at 1 hpi. The infected cells were incubated at 4°C for the attachment assay (Fig 3C and 3D) and at 37°C for the entry assay (Fig 3E and 3F). There were no significant differences in viral gDNA levels between *Arc* knockdown and control cells at the attachment step for PRV and HSV-1, whereas the levels of Ctrl cells with heparin-treated virus, used as a positive control, were significantly reduced for both PRV (Fig 3C) and HSV-1 (Fig 3D). Furthermore, *Arc* knockdown did not affect the entry of PRV (Fig 3E) or HSV-1 (Fig 3F).

As a result, *Arc* knockdown did not affect the viral attachment and entry into cells; thus, it is hypothesized that Arc may be involved in other processes in viral lifecycle, such as the intracytoplasmic translocation, entry into nucleus, and viral genome replication.

### Arc interacts with the viral major capsid protein VP5

**Fig 4:**
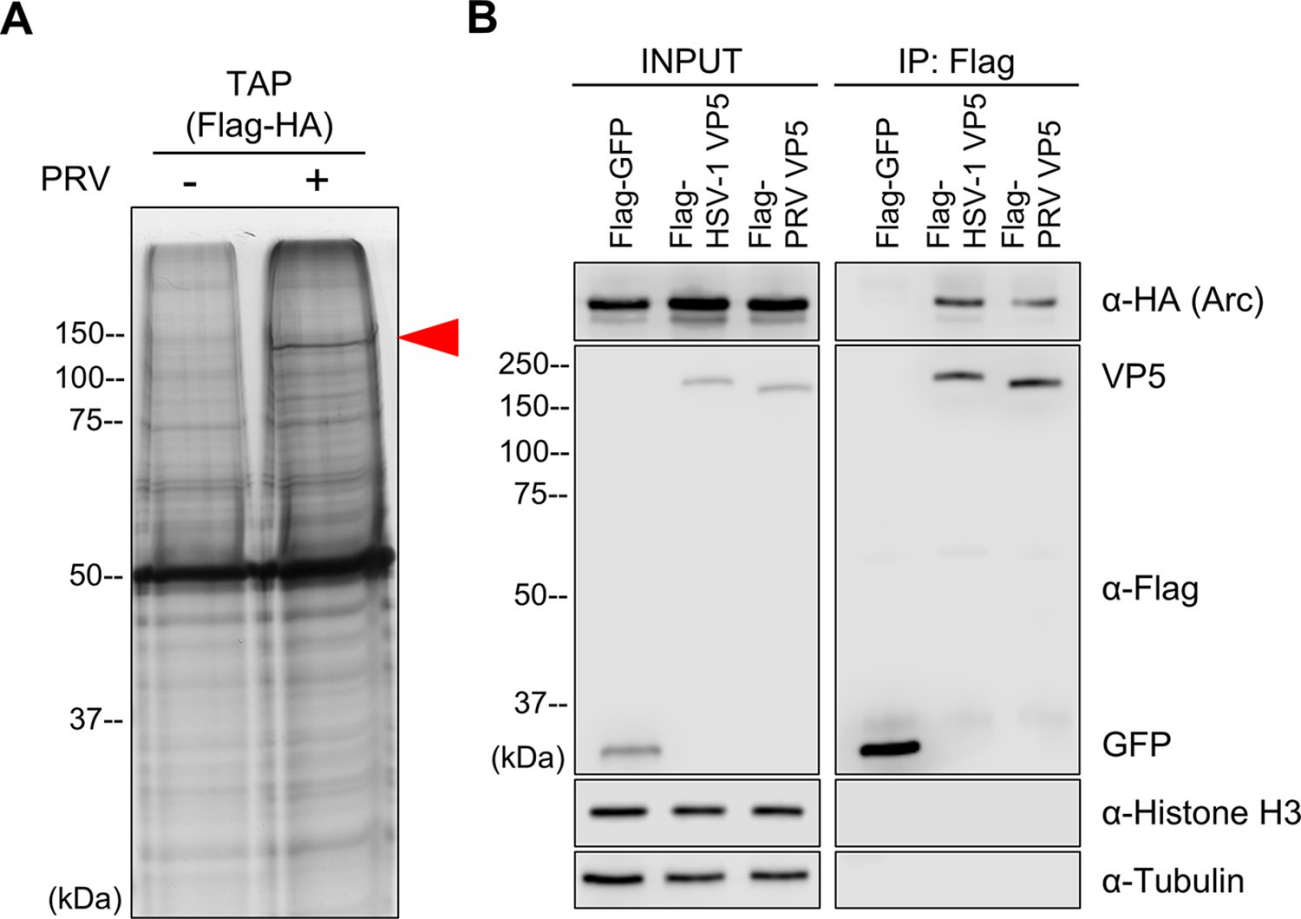
Arc interacts with the viral major capsid proteins VP5. (A) FLAG- and HA-tagged Arc expression plasmid was transfected into *Arc*-knockout HEK293T cells. At 24 h post-transfection, PRV was inoculated to the transfected *Arc*-knockout HEK293T cells at MOI of 5. The PRV infected or mock infected cell lysates were collected at 24 hpi, and Arc interacted proteins were purified by TAP assay and separated by SDS-PAGE. The specific band on PRV infected cells (red arrowhead) were detected by silver staining. (B) *Arc*-knockout HEK293T cells were co-transfected with the plasmids expressing HA-Arc and either Flag-GFP, Flag-PRV VP5, or Flag-HSV-1 VP5. At 24 h post-transfection, cell lysates were collected, and HA-Arc, Flag-VP5, Flag-GFP, tubulin and histone proteins were detected by western blotting.

To investigate the effect of Arc upregulation on alphaherpesvirus propagation, we searched for viral factors that interact with Arc. We transfected the plasmids encoding FLAG- and HA-tagged Arc into *Arc*-knockout HEK293T cells, and the transfected cells were infected with PRV at 24 h post-transfection. The intracellular proteins in PRV-infected and uninfected cells were purified using the TAP assay. Then, we performed SDS-PAGE and silver staining for each sample. A specific band of approximately 150 kDa was identified only in infected cells (Fig 4A). This specific band was analyzed and identified by mass spectrometry, which revealed the major capsid protein, VP5. To confirm the protein–protein interaction of Arc and PRV VP5 and to validate that of Arc and HSV-1 VP5, we performed co-IP by co-transfection of the plasmid encoding each viral FLAG-tagged VP5 and HA-tagged Arc into HEK293T cells. Co-IP analysis revealed an interaction between HA-tagged Arc and Flag-tagged VP5 from PRV or HSV-1, whereas no interaction was observed between HA-tagged Arc and Flag-tagged GFP, which was used as a control. Furthermore, nonspecific binding of intracellular proteins, such as tubulin and histone, was not detected in any of the pull-down samples (Fig 4B). In conclusion, the interaction between major capsid proteins VP5 and Arc is common phenomena among HSV-1 and PRV.

## Discussion

Here, we demonstrated that Arc upregulation occurred during PRV and HSV-1 replication and was induced by the PRV early gene EP0 and the HSV-1 homologue ICP0. Knockdown of Arc affects the viral proliferation in the early stage of viral infection in 8 hpi, but it did not prevent the viral attachment and entry into human neuronal cells. Arc interacted with PRV- and HSV-1-VP5, which are major capsid proteins.

We identified ICP0 and EP0 as one of the factors that upregulate Arc expression, which was upregulated at approximately 4 hpi (Fig 1A and 1C). ICP0 and EP0 are the early genes in HSV-1 and PRV, and their proteins are expressed in 2–4 hpi, indicating that the timing of Arc up-regulation matched the timing of ICP- or EP0-expression [23, 26]. ICP0 has the ability to trans-activate the expression of various host proteins, including ERVs in HSV-1 infection [27, 28]. The RING finger domain is highly conserved in the alphaherpesvirus ICP0 homologue, including EP0, and the RING finger domain functions as a trans-activator in viral infection; therefore, its nature is thought to be common among alphaherpesviruses [29]. It remains unclear how ICP0 homologues upregulated Arc expression, but considering the respective properties of EP0 and ICP0, we can provide some hypotheses. EP0 can recognize the TATA box and transcribe the downstream genes of the TATA box [30]. Two regions are described as Arc promoters: one is a critical synaptic activity-responsive element located >5 kb upstream of Arc, and the other is a minimal promoter located near Arc [31]. As the minimal promoter contains a TATA box, EP0 may indiscriminately upregulate Arc expression. Furthermore, Arc was originated from Ty3/Gypsy family which is one of the LTR-retroelement groups [14]. The expressions of HERV-K and HERV-W, whose elements are part of the LTR group, were upregulated by HSV-1 factors such as ICP0; however, the detailed mechanisms of this upregulation remain unclear [27, 32, 33]. Although the interactions between ICP0 and the promoters is unclear, EP0 and ICP0 may induce Arc through pathways involved in the expression of LTR retrotransposons. The Arc protein showed different expression levels in virus-infected cells and in the EP0 and ICP0 overexpression systems (Figs 1 and 2). It is possible that the enhancement or repression of various cellular pathways by viral infection led to differences in expression levels between infected and ICP0- or EP0-overexpressing cells. Arc expression is induced by various events, such as the brain-derived neurotrophic factor and metabotropic glutamate receptor activation, growth factor stimulation by NGF and EGF, cellular stresses including heat shock responses, upregulation of the MAPK upstream pathways, and Ca^2+^ intracellular signaling cascades [34, 35]. In addition to the typical transcriptional regulators observed in this study, various viral factors are partially involved in the transcriptional regulation of HSV-1 and PRV. Arc expression is thought to be considerably enhanced not only by the overexpression of ICP0 or EP0, but also by the interaction of various viral factors due to viral infection.

Besides, the potential involvement of cellular Arc capsids should not be overlooked. The effect of *Arc* knockdown on HSV-1 and PRV infection was observed within 8 hpi (Fig 3A and 3B), which roughly coincides with the time at which Arc expression increases after infection; thus, this phenomenon appears to be independent. The Arc protein, when overexpressed in cultured cells, assembles into a viral capsid-like structure and disseminates Arc mRNA to surrounding cells by incorporating Arc mRNA within the Arc capsid [13]. As it has been reported that viral infection occurs heterogeneously in relation to the cell state and the virus itself [36], it is possible that the Arc capsid is released from cells that have been infected early, and viral infection is assisted by Arc expression in the surrounding cells. For instance, it has been reported that human cytomegalovirus, which belongs to the Herpesviridae family, changes the cellular environment around infected cells using extracellular vesicles [37]. Furthermore, even in a single cell, early Arc expression due to a prior alphaherpes virus infection may facilitate subsequent alphaherpes viral infection. Although we were unable to evaluate these effects because we used a single-cycle infection system, future studies should confirm the presence or absence of capsid formation and its effects.

The effect of upregulated Arc expression on the HSV-1 life cycle was unclear in the previous study. As we identified VP5 as a viral protein that interacts with Arc in this study, we provide new insights into the relationship between Arc and viral infections. HSV-1 entry into cells can occur via two routes: envelope membrane fusion with the plasma membrane or endocytosis. In each route, capsids utilize microtubules, leading to nuclear translocation. In the current study, no significant differences were observed in cell-surface attachment or entry following *Arc* knockdown. This suggests that Arc can influence the viral step after cellular internalization, such as the microtubule-mediated intracytosolic transport, nuclear import, or viral genome replication. VP5, which interacts with Arc in this study, is a key capsid protein in herpesviruses [38, 39]. Movement of the HSV-1 capsid to the nucleus is facilitated by VP5 and UL36, UL37, which engage dynein motors and other accessory proteins [40, 41]. Arc interact with microtubules, where dynein and kinesin, the motor proteins carrying herpes virus, are expressed, and trigger changes in the cytoskeleton, potentially affecting transport along microtubules [42–44]. Based on these suggestions, Arc may affect the viral intracellular transport post entry; however, further studies are necessary to elucidate the role of Arc in the nuclear migration of alphaherpesviruses. In the present study, the possibility of specific binding between VP5 and Arc, rather than nonspecific aggregation, was suggested by attempting to detect tubulin and histone proteins. However, considering that VP5 is a structural protein and interacts with various viral proteins in capsid assembly [45–47], questions remain as to whether its interaction with Arc is maintained during capsid assembly, and whether binding is preserved even after tegumentation. Further investigation, including detailed analyses of the localization of Arc and VP5 proteins at various stages of viral replication, will be necessary to address these issues.

*In vivo* studies suggest that mutations, loss, or downregulated expression of Arc is also associated with the onset of neurological disorders such as autism, depression, and schizophrenia [48–51]. Arc is also required for the expression of neuronal activity-dependent amyloid beta, suggesting that high Arc expression is associated with the pathogenic mechanism of Alzheimer’s disease (AD) [52]. Reactivation of HSV-1 after latent infection and entry into the CNS are considered risk factors for AD [53]. ICP0, identified as an Arc expression inducer in this study, is encoded in the region of the latency-associated transcript, which is important for HSV-1 latent neuronal infection, and ICP0 expression is maintained during latent infection in neurons [54]. Overall, Arc upregulation by HSV-1 infection may be a risk factor for the induction of neuronal disorders, and this study will provide a stepping stone to elucidate the mechanisms underlying the onset of neurological diseases related to HSV-1 infection.

One concern throughout the present is that experiments were conducted using HEK293T and SH-SY5Y cells, which are human-derived cultured cells. While it is well known that PRV can infect cultured human cells, under natural conditions, PRV is a virus that causes Aujeszky’s disease when it infects pigs, and typical PRV strains do not exhibit pathogenicity in humans. Therefore, we cannot rule out the possibility that cells from a non-natural host may have affected the viral replication and intracellular dynamics. In the future, using porcine-derived cultured cells to investigate the effects of Arc may be necessary.

In this study, we provide evidence that PRV infection induces the expression of the host neuronal plasticity-related protein Arc, which is derived from the retroelement, a common phenomenon among alphaherpesviruses. It has also been suggested that the Arc proteins upregulated by the ICP0 homologue can interact with VP5, and *Arc* knockdown negatively affects the viral infection cycle after intracellular entry. Further studies are needed to investigate in detail which steps of viral lifecycle the interaction between Arc and VP5 contributes to after entry. However, this insight of a relationship between Arc and alphaherpesvirus may contribute to the mechanism underlying the neurological disorders associated with herpesvirus.

## Supporting information

S1 Fig Morphological differences between non-differentiated and differentiated SH-SY5Y cells. Images of SH-SY5Y cells before (A) and after differentiation (B). Scale bar indicates 50 μm.
(PDF)

S1 Raw data Mass spectrometry of VP5. (PDF)

S1 Raw images Original uncropped and blot/gel images. (PDF)
