# Supplementary figures and images for: Neuron-associated retroelement-derived protein Arc/Arg3.1 assists in the early stages of alphaherpesvirus infection in human neuronal cells
Source: PLoS One. 2024 Dec 12;19(12):e0314980. doi: 10.1371/journal.pone.0314980 (PMC11637343; doi:10.1371/journal.pone.0314980)

**A**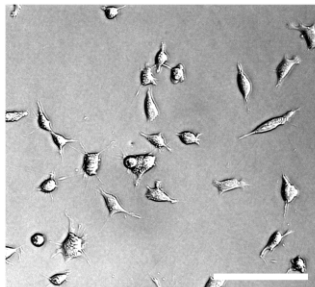**B**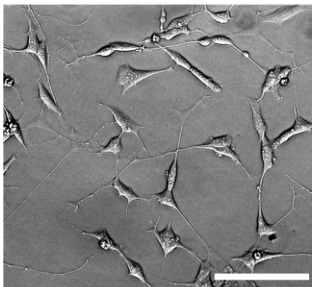

Supplement: S1 Fig — Images of SH-SY5Y cells before (A) and after differentiation (B). Scale bar indicates 50 μm. (PDF) [file pone.0314980.s001.pdf]
