# Supplementary material for: Neuron-associated retroelement-derived protein Arc/Arg3.1 assists in the early stages of alphaherpesvirus infection in human neuronal cells
Source: PLoS One. 2024 Dec 12;19(12):e0314980. doi: 10.1371/journal.pone.0314980 (PMC11637343; doi:10.1371/journal.pone.0314980)
Supplement: S1 Raw data — (PDF) [file pone.0314980.s002.pdf]

Results

Sample Name		Protein	gene	MW	Score	Peptide	Coverage	Accession	*Note**
TAP_Arc-PRV _150kDa	1	Major capsid protein [Suid herpesvirus 1 (Pseudorabies virus)]	MCP	145,938	632	17	17	Q00705	*MCP_SUHVS*

Note : Accession in Mascot Search Results*

TAP_Arc-PRV_150kDa- 1 **Major capsid protein [Suid herpesvirus 1 (Pseudorabies virus)]**

Mass: 145938 Score: 632 Matches: 17(4) Sequences: 17(4) emPAI: 0.10

Query	Observed	Mr(expt)	Mr(calc)	Delta	Miss	Score	Expect	Rank	Unique	Peptide	
152	484.81	967.61	967.58	0.0249	0	27	1.90E+00	1	U	R.LAPATVAAVR.G	
190	531.33	1060.64	1060.62	0.0188	0	40	1.30E-01	1	U	R.LVFLEALEK.R	
194	539.25	1076.49	1076.49	0.0018	0	34	4.50E-01	1	U	K.AVSNMDDVAR.Y	
195	540.81	1079.60	1079.57	0.0323	0	33	4.80E-01	1	U	K.FFTPAEAVAK.S	
203	580.84	1159.66	1159.63	0.0307	0	59	1.50E-03	1	U	R.VACAALVSELK.R	+Carbamidomethyl(C)
227	634.85	1267.69	1267.64	0.0406	0	84	4.20E-06	1	U	R.FATENVLFAEK.A	
233	639.34	1276.67	1276.64	0.0257	0	29	1.50E+00	1	U	R.NVQAVLDSFER.G	
258	458.93	1373.76	1373.76	-0.003	0	17	2.10E+01	1	U	R.NVGGGLVHNRPVR.N	
275	507.27	1518.80	1518.76	0.0421	0	16	2.60E+01	2	U	R.HAGSFAPTPGLPDPR.T	
279	531.96	1592.87	1592.83	0.0387	0	41	6.70E-02	1	U	R.TESGGGLHLQLTQPR.A	
284	836.95	1671.89	1671.86	0.032	0	59	1.10E-03	1	U	R.LSDLVNCTAPSVAVAR.M	+Carbamidomethyl(C)
290	582.30	1743.88	1743.89	-0.019	0	20	9.50E+00	1	U	R.TPLGAEEHFAQYLIR.D	
291	873.01	1744.01	1743.96	0.0585	0	49	9.00E-03	1	U	K.APPLSLLAPFTLYEGR.L	
294	897.45	1792.89	1792.83	0.0552	0	32	5.70E-01	1	U	R.ADENTLSYALMAGYFK.M	
296	606.97	1817.89	1817.85	0.0388	0	24	3.10E+00	1	U	R.DGPHPADQPVHNYMIK.R	
301	633.67	1898.00	1897.95	0.0464	0	38	1.30E-01	1	U	R.YLLGGEPAPDDGKPVGSAR.V	
318	611.31	2441.22	2441.21	0.0174	0	37	1.10E-01	1	U	R.AAAQHVPCVPHFLGANYYATVR.Q	+Carbamidomethyl(C)

MATRIX SCIENCE MASCOT Search Results

Protein View: MCP_SUHVS

Major capsid protein OS=Suid herpesvirus 1 (strain Indiana S) OX=31522 GN=MCP PE=3 SV=1

**Database:**	SwissProt
**Score:**	632
**Monoisotopic mass (M_r):**	145844
**Calculated pI:**	6.03
**Taxonomy:**	Suid herpesvirus 1 (strain Indiana S)

Sequence similarity is available as an NCBI BLAST search of MCP_SUHVS against nr.

Search parameters

**MS data file:**	mas87B.tmp
**Enzyme:**	Trypsin: cuts C-term side of KR unless next residue is P.
**Variable modifications:**	Carbamidomethyl (C), Oxidation (M)

Protein sequence coverage: 17%

Matched peptides shown in ***bold red***.

1 MERPAILPSG QILSNIEVHS HRALFDIFKR FRSDDNNLYG AEFDALLGTY
51 CSTLSLVRFL ELGLSVACVC TKFPELSYVA EGTIQFEVQQ PMIAR**DGPHP**
101 **ADQPVHNYMI** **K**RLDRRSLNA AFSIAVEALG LISGENLDGT HISSAMRLRA
151 IQQLAR**NVQA** **VLDSFER**GTA DQMLRVLMEK **APPLSLLAPF** **TLYEGR**LADR
201 **VACAALVSEL** **K**RRVRDDTFF LTKHERNKDA VLDR**LSDLVN** **CTAPSVAVAR**
251 MTHADTQGRP VDGVLVTTAG VRQRLLHHVL TLADTHADVP VTYGEMVIAN
301 TNLVTALVMG K**AVSNMDDVA** **RYLLGGEPAP** **DDGKPVGSAR** VRADLVVVGD
351 R**LVFLEALEK** RVYQATQVPY PLVGNLDVTF VMPLGVFKPA ADRYAR**HAGS**
401 **FAPTPGLPDP** **R**THPPRAVHF FNKDGVPCHV TFEHAMGTLC HPSFLDVDAT
451 LAALRQEPAE VQCAFGAYVA DARPDALVGL MQRFLEEWPG MMPVRPRWAA
501 PAAADQLLAP GNADLRLELH PAFDFFVAPE VDVPGPFAVP QVMGQVRAMP
551 RIINGNIPLA LCPVDFRDAR GFELSVDRHR **LAPATVAAVR** GAFRDANYPM
601 VFYIIEAVIH GSERTFCALA RLVAQCIQSY WRNTHNAAFV NNFYMVMYIN
651 TYLGNGELPE DCAAVYKDLL EHVHALRRLI GEFTLPGDPL GNQPQEELNH
701 ALADATLLPP LIWDCDPILY RDGLAERLPE LRVNGAHFQH ILWVEMAQVN
751 FR**NVGGGLVH** **NRPVR**NENQP LHPHHDAEWS VLSKIYYYAV VPAFSRGNCC
801 TMGVRYDRVY QLVQTMVVPE TDEEVGTDDP RHPLHPRNLV PNSLNVLFHN
851 ACVAVDADAM LILQETVTNM AERTTPLLAS VAPDAGMATV ATRDMRTHDG
901 SLHHGLLMMA YQPNDATLLE GAFFYPAPVN ALFACADHLG AMRDVGAEVR
951 **AAAQHVPCVP** **HFLGANYYAT** **VR**QPVAQHAA QSR**ADENTLS** **YALMAGYFK**M
1001 SPVAFTHQLR RQLHPGFALT VVRQDR**FATE** **NVLFAEK**ASE SYFMGQMQVA
1051 R**TESGGGLHL** **QLTQPR**ANVD LGVGFTAAYA AAALRAPVTD MGNLPQNLFA
1101 TRGAPPMLDA DADDYLRRTV NAGNRLAPVP VFGQMLPQVP AGLARGQQSV
1151 CEFIATPVSV DLAYFRRACN PRGRAAGEVH GEEGLMFDHS HADPAHPHRA
1201 TANPWASQRH SYADRLYNGQ YNMSGPAYSP CFK**FFTPAEA** **VAK**SRGLARL
1251 IADTGAAASP TSNGEYQFKR PVGAGELVED PCALFQEAYP PLCASDSALL
1301 R**TPLGAEEHF** **AQYLIR**DESP LKGCFQHASA

Unformatted sequence string: 1330 residues (for pasting into other applications).

Sort by ◉ residue number ○ increasing mass ○ decreasing mass
Show ◉ matched peptides only ○ predicted peptides also

Query	Start - End	Observed	Mr(expt)	Mr(calc)	Delta	M	Score	Expect	Rank	U	Peptide
296	96 - 111	606.9708	1817.8907	1817.8519	0.0388	0	24	3.1	1	U	R.DGPHPADQPVHNYMIK.R
233	157 - 167	639.3407	1276.6668	1276.6412	0.0257	0	29	1.5	1	U	R.NVQAVLDSFER.G
291	181 - 196	873.0145	1744.0144	1743.9559	0.0585	0	49	0.009	1	U	K.APPLSLLAPFTLYEGR.L
203	201 - 211	580.8362	1159.6578	1159.6271	0.0307	0	59	0.0015	1	U	R.VACAALVSELK.R + Carbamidomethyl (C)
284	235 - 250	836.9540	1671.8934	1671.8614	0.0320	0	59	0.0011	1	U	R.LSDLVNCTAPSVAVAR.M + Carbamidomethyl (C)
194	312 - 321	539.2542	1076.4938	1076.4920	0.0018	0	34	0.45	1	U	K.AVSNMDDVAR.Y
301	322 - 340	633.6739	1897.9998	1897.9534	0.0464	0	38	0.13	1	U	R.YLLGGEPAPDDGKPVGSAR.V
190	352 - 360	531.3251	1060.6356	1060.6168	0.0188	0	40	0.13	1	U	R.LVFLEALEK.R
275	397 - 411	507.2739	1518.8000	1518.7579	0.0421	0	16	26	2	U	R.HAGSFAPTPGLPDPR.T
152	581 - 590	484.8105	967.6064	967.5815	0.0249	0	27	1.9	1	U	R.LAPATVAAVR.G
258	753 - 765	458.9278	1373.7615	1373.7640	-0.0025	0	17	21	1	U	R.NVGGGLVHNRPVR.N
318	951 - 972	611.3132	2441.2237	2441.2063	0.0174	0	37	0.11	1	U	R.AAAQHVPCVPHFLGANYYATVR.Q + Carbamidomethyl (C)
294	984 - 999	897.4520	1792.8894	1792.8342	0.0552	0	32	0.57	1	U	R.ADENTLSYALMAGYFK.M
227	1027 - 1037	634.8500	1267.6855	1267.6448	0.0406	0	84	4.2e-06	1	U	R.FATENVLFAEK.A
279	1052 - 1066	531.9625	1592.8658	1592.8271	0.0387	0	41	0.067	1	U	R.TESGGGLHLQLTQPR.A
195	1234 - 1243	540.8060	1079.5974	1079.5651	0.0323	0	33	0.48	1	U	K.FFTPAEAVAK.S

Query	Start - End	Observed	Mr(expt)	Mr(calc)	Delta	M	Score	Expect	Rank	U	Peptide
290	1302 - 1316	582.2991	1743.8754	1743.8944	-0.0190	0	20	9.5	1	U	R.TPLGAEEHFAQYLIR.D

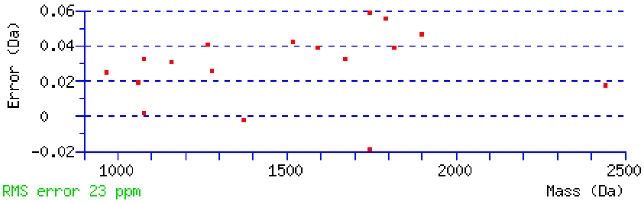

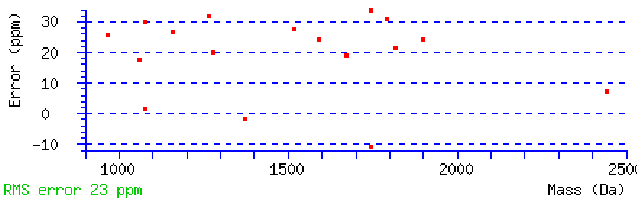

```
ID   MCP_SUHVS               Reviewed;        1330 AA.
AC   Q00705;
DT   01-APR-1993, integrated into UniProtKB/Swiss-Prot.
DT   01-APR-1993, sequence version 1.
DT   12-AUG-2020, entry version 59.
DE   RecName: Full=Major capsid protein {ECO:0000255|HAMAP-Rule:MF_04016};
DE            Short=MCP {ECO:0000255|HAMAP-Rule:MF_04016};
GN   Name=MCP {ECO:0000255|HAMAP-Rule:MF_04016};
OS   Suid herpesvirus 1 (strain Indiana S) (SuHV-1) (Pseudorabies virus (strain
OS   Indiana S)).
OC   Viruses; Duplodnaviria; Heunggongvirae; Peploviricota; Herviviricetes;
OC   Herpesvirales; Herpesviridae; Alphaherpesvirinae; Varicellovirus.
OX   NCBI_TaxID=31522;
OH   NCBI_TaxID=9823; Sus scrofa (Pig).
RN   [1]
RP   NUCLEOTIDE SEQUENCE [GENOMIC DNA].
RX   PubMed=1718089; DOI=10.1016/0042-6822(91)90753-x;
RA   Yamada S., Imada T., Watanabe W., Honda Y., Nakajima-Iijima S., Shimizu Y.,
RA   Sekikawa K.;
RT   "Nucleotide sequence and transcriptional mapping of the major capsid
RT   protein gene of pseudorabies virus.";
RL   Virology 185:56-66(1991).
CC   -!- FUNCTION: Self-assembles to form an icosahedral capsid with a T=16
CC       symmetry, about 200 nm in diameter, and consisting of 150 hexons and 12
CC       pentons (total of 162 capsomers). Hexons form the edges and faces of
CC       the capsid and are each composed of six MCP molecules. In contrast, one
CC       penton is found at each of the 12 vertices. Eleven of the pentons are
CC       MCP pentamers, while the last vertex is occupied by the portal complex.
CC       The capsid is surrounded by a layer of proteinaceous material
CC       designated the tegument which, in turn, is enclosed in an envelope of
CC       host cell-derived lipids containing virus-encoded glycoproteins.
CC       {ECO:0000255|HAMAP-Rule:MF_04016}.
CC   -!- SUBUNIT: Homomultimer. Makes the hexons and eleven out of twelve
CC       pentons. Interacts with triplex proteins 1/TRX1 and 2/TRX2; adjacent
CC       capsomers are linked together in groups of three by triplexes,
CC       heterotrimeric complexes composed of one molecule of TRX1 and two
CC       molecules of TRX2. Interacts with scaffold protein; this interaction
CC       allows efficient MCP transport to the host nucleus. Interacts with
CC       capsid vertex component 2/CVC2. Interacts with the small capsomere-
CC       interacting protein/SCP. {ECO:0000255|HAMAP-Rule:MF_04016}.
CC   -!- SUBCELLULAR LOCATION: Virion {ECO:0000255|HAMAP-Rule:MF_04016}. Host
CC       nucleus {ECO:0000255|HAMAP-Rule:MF_04016}.
CC   -!- SIMILARITY: Belongs to the herpesviridae major capsid protein family.
CC       {ECO:0000255|HAMAP-Rule:MF_04016}.
CC   ---------------------------------------------------------------------------
CC   Copyrighted by the UniProt Consortium, see https://www.uniprot.org/terms
CC   Distributed under the Creative Commons Attribution (CC BY 4.0) License
CC   ---------------------------------------------------------------------------
DR   PIR; A40777; VCBES5.
DR   DIP; DIP-62100N; -.
DR   PRIDE; Q00705; -.
DR   GO; GO:0042025; C:host cell nucleus; IEA:UniProtKB-SubCell.
DR   GO; GO:0039622; C:T=16 icosahedral viral capsid; IEA:UniProtKB-UniRule.
DR   GO; GO:0005198; F:structural molecule activity; IEA:UniProtKB-UniRule.
DR   HAMAP; MF_04016; HSV_MCP; 1.
DR   InterPro; IPR000912; Herpes_MCP.
DR   InterPro; IPR023233; Herpes_MCP_upper_sf.
DR   Pfam; PF03122; Herpes_MCP; 1.
DR   PRINTS; PR00235; HSVCAPSIDMCP.
DR   SUPFAM; SSF103417; SSF103417; 1.
PE   3: Inferred from homology;
KW   Capsid protein; Host nucleus; T=16 icosahedral capsid protein; Virion.
FT   CHAIN           1..1330
FT                   /note="Major capsid protein"
FT                   /id="PRO_0000115708"
SQ   SEQUENCE   1330 AA;  145938 MW;  4E228145F773A522 CRC64;
     MERPAILPSG QILSNIEVHS HRALFDIFKR FRSDDNNLYG AEFDALLGTY CSTLSLVRFL
     ELGLSVACVC TKFPELSYVA EGTIQFEVQQ PMIARDGPHP ADQPVHNYMI KRLDRRSLNA
     AFSIAVEALG LISGENLDGT HISSAMRLRA IQQLARNVQA VLDSFERGTA DQMLRVLMEK
     APPLSLLAPF TLYEGRLADR VACAALVSEL KRRVRDDTFF LTKHERNKDA VLDRLSDLVN
     CTAPSVAVAR MTHADTQGRP VDGVLVTTAG VRQRLLHHVL TLADTHADVP VTYGEMVIAN
     TNLVTALVMG KAVSNMDDVA RYLLGGEPAP DDGKPVGSAR VRADLVVVGD RLVFLEALEK
     RVYQATQVPY PLVGNLDVTF VMPLGVFKPA ADRYARHAGS FAPTPGLPDP RTHPPRAVHF
     FNKDGVPCHV TFEHAMGTLC HPSFLDVDAT LAALRQEPAE VQCAFGAYVA DARPDALVGL
     MQRFLEEWPG MMPVRPRWAA PAADQLLAP GNADLRLELH PAFDFFVAPE VDVPGPFAVP
     QVMGQVRAMP RIINGNIPLA LCPVDFRDAR GFELSVDRHR LAPATVAAVR GAFRDANYPM
     VFYIIEAVIH GSERTFCALA RLVAQCIQSY WRNTHNAAFV NNFYMVMYIN TYLGNGELPE
     DCAAVYKDLL EHVHALRRLI GEFTLPGDPL GNQPQEELNH ALADATLLPP LIWDCDPILY
     RDGLAERLPE LRVNGAHFQH ILWVEMAQVN FRNVGGGLVH NRPVRNENQP LHPHHDAEWS
     VLSKIYYYAV VPAFSRGNCC TMGVRYDRVY QLVQTMVVPE TDEEVGTDDP RHPLHPRNLV
     PNSLNVLFHN ACVAVDADAM LILQETVTNM AERTTPLLAS VAPDAGMATV ATRDMRTHDG
```

```
SLHHGLLMMA YQPNDATLLE GAFFYPAPVN ALFACADHLG AMRDVGAEVR AAAQHVPCVP
HFLGANYYAT VRQPVAQHAA QSRADENTLS YALMAGYFKM SPVAFTHQLR RQLHPGFALT
VVRQDRFATE NVLFAEKASE SYFMGQMQVA RTESGGGLHL QLTQPRANVD LGVGFTAAYA
AAALRAPVTD MGNLPQNLFA TRGAPPMLDA DADDYLRRTV NAGNRLAPVP VFGQMLPQVP
AGLARGQQSV CEFIATPVSV DLAYFRRACN PRGRAAGEVH GEEGLMFDHS HADPAHPHRA
TANPWASQRH SYADRLYNGQ YNMSGPAYSP CFKFFTPAEA VAKSRGLARL IADTGAAASP
TSNGEYQFKR PVGAGELVED PCALFQEAYP PLCASDSALL RTPLGAEEHF AQYLIRDESP
LKGCFQHASA
```

Mascot Search Results

User	: JPROS
Email	: pro@jbios.co.jp
Search title	: D:\JPROS\N094\150kDa_8micro.wiff (sample number 1)
MS data file	: mas87B.tmp
Database	: SwissProt 2021_03 (565254 sequences; 203850821 residues)
Timestamp	: 3 Jul 2021 at 09:27:52 GMT
Protein hits	: MCP_SUHVS Major capsid protein OS=Suid herpesvirus 1 (strain Indiana S) OX=31522 GN=MCP PE=3 SV=1
	K2C1_PANTR Keratin, type II cytoskeletal 1 OS=Pan troglodytes OX=9598 GN=KRT1 PE=2 SV=1
	K1C9_HUMAN Keratin, type I cytoskeletal 9 OS=Homo sapiens OX=9606 GN=KRT9 PE=1 SV=3
	TRYP_PIG Trypsin OS=Sus scrofa OX=9823 PE=1 SV=1
	K22E_HUMAN Keratin, type II cytoskeletal 2 epidermal OS=Homo sapiens OX=9606 GN=KRT2 PE=1 SV=2
	K1C10_HUMAN Keratin, type I cytoskeletal 10 OS=Homo sapiens OX=9606 GN=KRT10 PE=1 SV=6
	K2C6B_HUMAN Keratin, type II cytoskeletal 6B OS=Homo sapiens OX=9606 GN=KRT6B PE=1 SV=5
	K2C8_MOUSE Keratin, type II cytoskeletal 8 OS=Mus musculus OX=10090 GN=Krt8 PE=1 SV=4
	K2C73_RAT Keratin, type II cytoskeletal 73 OS=Rattus norvegicus OX=10116 GN=Krt73 PE=1 SV=1
	K2CO_CHICK Keratin, type II cytoskeletal cochleal OS=Gallus gallus OX=9031 PE=2 SV=1
	K2C7_BOVIN Keratin, type II cytoskeletal 7 OS=Bos taurus OX=9913 GN=KRT7 PE=2 SV=1
	PEX1_HUMAN Peroxisome biogenesis factor 1 OS=Homo sapiens OX=9606 GN=PEX1 PE=1 SV=1

Mascot Score Histogram

Ions score is -10*Log(P), where P is the probability that the observed match is a random event.
Individual ions scores > 41 indicate identity or extensive homology(p<0.05).
Protein scores are derived from ions scores as a non-probabilistic basis for ranking protein hits.

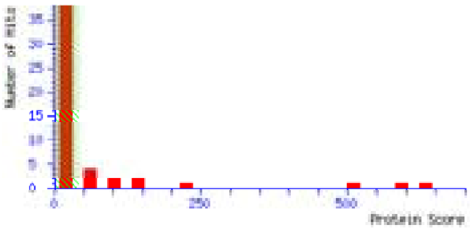

Peptide Summary Report

Format As | Peptide Summary | Help
Significance threshold p< 0.05 | Max. number of hits AUTO
Standard scoring ◉ MudPIT scoring ○ | Display non-significant matches ☑ | Show sub-sets 0
Show pop-ups ◉ Suppress pop-ups ○ | Sort unassigned Decreasing Score | Require bold red ☐
Preferred taxonomy All entries

Select All | Select None | Search Selected | ☐ **Error tolerant**

1. MCP_SUHVS **Mass:** 145844 **Score:** 632 **Matches:** 17(4) **Sequences:** 17(4) **emPAI:** 0.10
Major capsid protein OS=Suid herpesvirus 1 (strain Indiana S) OX=31522 GN=MCP PE=3 SV=1
☐ Check to include this hit in error tolerant search

	Query	Observed	Mr(expt)	Mr(calc)	Delta	Miss	Score	Expect	Rank	Unique	Peptide
☑	152	484.8105	967.6064	967.5815	0.0249	0	27	1.9	1	U	R.LAPATVAAVR.G
☑	190	531.3251	1060.6356	1060.6168	0.0188	0	40	0.13	1	U	R.LVFLEALEK.R
☑	194	539.2542	1076.4938	1076.4921	0.0018	0	34	0.45	1	U	K.AVSNMDDVAR.Y
☑	195	540.8060	1079.5974	1079.5651	0.0323	0	33	0.48	1	U	K.FFTPAEAVAK.S
☑	203	580.8362	1159.6578	1159.6271	0.0307	0	59	0.0015	1	U	R.VACAALVSELK.R + Carbamidomethyl (C)
☑	227	634.8500	1267.6855	1267.6448	0.0406	0	84	4.2e-06	1	U	R.FATENVLFAEK.A
☑	233	639.3407	1276.6668	1276.6412	0.0257	0	29	1.5	1	U	R.NVQAVLDSFER.G
☑	258	458.9278	1373.7615	1373.7640	-0.0025	0	17	21	1	U	R.NVGGGLVHNRPVR.N
	275	507.2739	1518.8000	1518.7579	0.0421	0	16	26	2	U	R.HAGSFAPTPGLPDPR.T
☑	279	531.9625	1592.8658	1592.8271	0.0387	0	41	0.067	1	U	R.TESGGGLHLQLTQPR.A
☑	284	836.9540	1671.8934	1671.8614	0.0320	0	59	0.0011	1	U	R.LSDLVNCTAPSVAVAR.M + Carbamidomethyl (C)
☑	290	582.2991	1743.8754	1743.8944	-0.0190	0	20	9.5	1	U	R.TPLGAEEHFAQYLIR.D
☑	291	873.0145	1744.0144	1743.9559	0.0585	0	49	0.009	1	U	K.APPLSLLAPFTLYEGR.L
☑	294	897.4520	1792.8894	1792.8342	0.0552	0	32	0.57	1	U	R.ADENTLSYALMAGYFK.M
☑	296	606.9709	1817.8907	1817.8519	0.0388	0	24	3.1	1	U	R.DGPHPADQPVHNYMIK.R
☑	301	633.6739	1897.9998	1897.9534	0.0464	0	38	0.13	1	U	R.YLLGGEPAPDDGKPVGSAR.V
☑	318	611.3132	2441.2237	2441.2063	0.0174	0	37	0.11	1	U	R.AAAQHVPCVPHFLGANYYATVR.Q + Carbamidomethyl (C)

2. K2C1_PANTR **Mass:** 65450 **Score:** 598 **Matches:** 22(7) **Sequences:** 15(7) **emPAI:** 0.46
Keratin, type II cytoskeletal 1 OS=Pan troglodytes OX=9598 GN=KRT1 PE=2 SV=1
☐ Check to include this hit in error tolerant search

	Query	Observed	Mr(expt)	Mr(calc)	Delta	Miss	Score	Expect	Rank	Unique	Peptide
☑	124	437.7633	873.5120	873.4920	0.0200	0	27	2.6	1	U	R.SLVNLGGSK.S
☑	125	437.7722	873.5297	873.4920	0.0378	0	(26)	3.7	1	U	R.SLVNLGGSK.S
	155	487.2774	972.5402	972.5240	0.0162	0	(18)	21	4		K.IEISELNR.V
☑	156	487.2835	972.5524	972.5240	0.0284	0	27	2.8	1		K.IEISELNR.V
	178	517.2678	1032.5211	1032.5087	0.0124	0	(13)	60	5	U	R.TLLEGEESR.M
	179	517.2799	1032.5453	1032.5087	0.0366	0	13	58	3	U	R.TLLEGEESR.M
	193	533.2699	1064.5252	1064.5138	0.0114	0	20	11	2	U	K.AQYEDIAQK.S

☑	200	563.2873	1124.5601	1124.5349	0.0251	0	36	0.28	1	U	K.AEAESLYQSK.Y
☑	205	590.3127	1178.6109	1178.5931	0.0178	0	(29)	1.4	1		K.YEELQITAGR.H
☑	206	590.3152	1178.6158	1178.5931	0.0227	0	44	0.048	1		K.YEELQITAGR.H
	233	639.3407	1276.6668	1276.7027	-0.0358	0	9	1.4e+02	4	U	K.LALDLEIATYR.T
☑	249	651.8764	1301.7383	1301.7078	0.0305	0	60	0.001	1		R.SLDLDSIIAEVK.A
☑	256	670.8534	1339.6923	1339.6619	0.0304	1	34	0.38	1	U	K.SKAEAESLYQSK.Y
☑	260	692.3712	1382.7279	1382.6830	0.0449	0	83	5.1e-06	1	U	K.SLNNQFASFIDK.V
☑	262	465.2602	1392.7587	1392.7249	0.0339	1	34	0.35	1	U	R.TNAENEFVTIKK.D
☑	263	465.2641	1392.7704	1392.7249	0.0456	1	(10)	92	1	U	R.TNAENEFVTIKK.D
☑	267	738.3922	1474.7699	1474.7416	0.0283	0	(10)	1.1e+02	1	U	K.WELLQQVDTSTR.T
☑	268	738.4035	1474.7924	1474.7416	0.0508	0	42	0.058	1	U	K.WELLQQVDTSTR.T
☑	269	738.4041	1474.7936	1474.7416	0.0520	0	(21)	8.6	1	U	K.WELLQQVDTSTR.T
☑	270	738.4212	1474.8278	1474.7780	0.0498	0	67	0.00019	1		R.FLEQQNQVLQTK.W
☑	281	546.9665	1637.8777	1637.8525	0.0251	1	42	0.053	1	U	K.SLNNQFASFIDKVR.F
☑	304	665.3436	1993.0090	1992.9693	0.0396	0	64	0.00034	1	U	R.THNLEPYFESFINNLR.R

Proteins matching the same set of peptides:
K2C1_HUMAN **Mass:** 65999 **Score:** 597 **Matches:** 22(7) **Sequences:** 15(7)
Keratin, type II cytoskeletal 1 OS=Homo sapiens OX=9606 GN=KRT1 PE=1 SV=6

---

3. K1C9_HUMAN **Mass:** 62027 **Score:** 506 **Matches:** 18(5) **Sequences:** 12(5) **emPAI:** 0.33
Keratin, type I cytoskeletal 9 OS=Homo sapiens OX=9606 GN=KRT9 PE=1 SV=3
☐ Check to include this hit in error tolerant search

	Query	Observed	Mr(expt)	Mr(calc)	Delta	Miss	Score	Expect	Rank	Unique	Peptide
☑	132	449.2245	896.4345	896.4062	0.0283	0	28	1.5	1	U	R.MTLDDFR.I
☑	133	449.2256	896.4366	896.4062	0.0304	0	(16)	22	1	U	R.MTLDDFR.I
	134	449.2385	896.4625	896.4062	0.0563	0	(2)	4.9e+02	4	U	R.MTLDDFR.I
☑	135	449.2388	896.4630	896.4062	0.0568	0	(18)	12	1	U	R.MTLDDFR.I
☑	188	530.8026	1059.5906	1059.5560	0.0345	0	36	0.34	1	U	K.TLLDIDNTR.M
	189	530.8027	1059.5908	1059.5560	0.0348	0	(24)	5.3	3	U	K.TLLDIDNTR.M
	191	533.2566	1064.4986	1064.4920	0.0066	0	30	1.1	2	U	K.STMQELNSR.L
	192	533.2695	1064.5244	1064.4920	0.0324	0	(9)	1.2e+02	6	U	K.STMQELNSR.L
	193	533.2699	1064.5252	1064.4920	0.0332	0	(8)	1.7e+02	8	U	K.STMQELNSR.L
☑	202	579.3089	1156.6032	1156.5836	0.0195	0	53	0.0063	1	U	R.QGVDADINGLR.Q
☑	208	595.8174	1189.6202	1189.6013	0.0189	0	40	0.12	1	U	R.QVLDNLTMEK.S
☑	278	793.8977	1585.7809	1585.7583	0.0226	0	25	2.8	1	U	K.VQALEEANNDLENK.I
☑	297	613.3321	1836.9746	1836.9581	0.0164	0	36	0.22	1	U	R.HGVQELEIELQSQLSK.K
☑	299	617.9910	1850.9510	1850.9196	0.0314	1	32	0.5	1	U	K.TLNDMRQEYEQLIAK.N
☑	314	767.0602	2298.1588	2298.1123	0.0465	1	59	0.00078	1	U	K.SDLEMQYETLQEELMALKK.N
☑	317	793.0802	2376.2189	2376.1808	0.0381	1	51	0.0048	1	U	R.LASYLDKVQALEEANNDLENK.I
☑	322	968.1565	2901.4476	2901.4032	0.0444	1	48	0.0071	1	U	K.NYSPYYNTIDDLKDQIVDLTVGNNK.T
☑	323	1088.8542	3263.5407	3263.5066	0.0342	0	69	5.2e-05	1	U	K.DIENQYETQITQIEHEVSSSGQEVQSSAK.E

---

4. TRYP_PIG **Mass:** 24394 **Score:** 211 **Matches:** 7(4) **Sequences:** 4(3) **emPAI:** 0.77
Trypsin OS=Sus scrofa OX=9823 PE=1 SV=1
☐ Check to include this hit in error tolerant search

	Query	Observed	Mr(expt)	Mr(calc)	Delta	Miss	Score	Expect	Rank	Unique	Peptide
☑	89	421.7710	841.5274	841.5022	0.0252	0	32	0.62	1	U	R.VATVSLPR.S
☑	90	421.7733	841.5320	841.5022	0.0298	0	(27)	1.8	1	U	R.VATVSLPR.S
	183	523.2802	1044.5459	1044.5564	-0.0105	0	(10)	1.3e+02	3	U	K.LSSPATLNSR.V
☑	184	523.2964	1044.5782	1044.5564	0.0219	0	45	0.04	1	U	K.LSSPATLNSR.V
☑	312	737.7190	2210.1350	2210.0967	0.0383	0	71	5.5e-05	1	U	R.LGEHNIDVLEGNEQFINAAK.I
☑	313	761.7499	2282.2277	2282.1729	0.0549	0	63	0.00031	1	U	K.IITHPNFNGNTLDNDIMLIK.L
☑	315	767.0713	2298.1920	2298.1678	0.0242	0	(59)	0.00085	1	U	K.IITHPNFNGNTLDNDIMLIK.L + Oxidation (M)

---

5. K22E_HUMAN **Mass:** 65393 **Score:** 156 **Matches:** 6(2) **Sequences:** 3(2) **emPAI:** 0.12
Keratin, type II cytoskeletal 2 epidermal OS=Homo sapiens OX=9606 GN=KRT2 PE=1 SV=2
☐ Check to include this hit in error tolerant search

	Query	Observed	Mr(expt)	Mr(calc)	Delta	Miss	Score	Expect	Rank	Unique	Peptide
	155	487.2774	972.5402	972.5240	0.0162	0	(18)	21	4		K.IEISELNR.V
	156	487.2835	972.5524	972.5240	0.0284	0	27	2.8	1		K.IEISELNR.V
☑	253	665.3858	1328.7570	1328.7187	0.0383	0	63	0.00046	1		R.NLDLDSIIAEVK.A
	268	738.4035	1474.7924	1474.7780	0.0144	0	(14)	38	2		R.FLEQQNQVLQTK.W
	269	738.4041	1474.7936	1474.7780	0.0156	0	(11)	74	3		R.FLEQQNQVLQTK.W
	270	738.4212	1474.8278	1474.7780	0.0498	0	67	0.00019	1		R.FLEQQNQVLQTK.W

---

6. K1C10_HUMAN **Mass:** 58792 **Score:** 133 **Matches:** 4(1) **Sequences:** 4(1) **emPAI:** 0.06
Keratin, type I cytoskeletal 10 OS=Homo sapiens OX=9606 GN=KRT10 PE=1 SV=6
☐ Check to include this hit in error tolerant search

	Query	Observed	Mr(expt)	Mr(calc)	Delta	Miss	Score	Expect	Rank	Unique	Peptide
☑	177	516.3094	1030.6042	1030.5910	0.0132	0	26	2.7	1	U	R.VLDELTLTK.A
☑	197	545.7855	1089.5564	1089.5237	0.0328	0	38	0.19	1	U	K.VTMQNLNDR.L
☑	259	691.3417	1380.6688	1380.6408	0.0280	0	56	0.0024	1	U	R.ALEESNYELEGK.I
☑	272	498.6046	1492.7919	1492.7270	0.0650	1	13	52	1	U	R.SQYEQLAEQNRK.D

---

7. K2C6B_HUMAN **Mass:** 60030 **Score:** 107 **Matches:** 3(2) **Sequences:** 2(2) **emPAI:** 0.13
Keratin, type II cytoskeletal 6B OS=Homo sapiens OX=9606 GN=KRT6B PE=1 SV=5
☐ Check to include this hit in error tolerant search

Query	Observed	Mr(expt)	Mr(calc)	Delta	Miss	Score	Expect	Rank	Unique	Peptide
205	590.3127	1178.6109	1178.5931	0.0178	0	(29)	1.4	1		K.YEELQITAGR.H
206	590.3152	1178.6158	1178.5931	0.0227	0	44	0.048	1		K.YEELQITAGR.H
253	665.3858	1328.7570	1328.7187	0.0383	0	63	0.00046	1		R.NLDLDSIIAEVK.A

---

8. K2C8_MOUSE **Mass:** 54531 **Score:** 83 **Matches:** 1(1) **Sequences:** 1(1) **emPAI:** 0.07
Keratin, type II cytoskeletal 8 OS=Mus musculus OX=10090 GN=Krt8 PE=1 SV=4
☐ Check to include this hit in error tolerant search

Query	Observed	Mr(expt)	Mr(calc)	Delta	Miss	Score	Expect	Rank	Unique	Peptide
260	692.3712	1382.7279	1382.7194	0.0085	1	83	5.1e-06	1	U	K.SLNNKFASFIDK.V

---

9. K2C73_RAT **Mass:** 60349 **Score:** 76 **Matches:** 4(1) **Sequences:** 2(1) **emPAI:** 0.06
Keratin, type II cytoskeletal 73 OS=Rattus norvegicus OX=10116 GN=Krt73 PE=1 SV=1
☐ Check to include this hit in error tolerant search

Query	Observed	Mr(expt)	Mr(calc)	Delta	Miss	Score	Expect	Rank	Unique	Peptide
233	639.3407	1276.6668	1276.7027	-0.0358	0	9	1.4e+02	4		K.LALDIEIATYR.K
268	738.4035	1474.7924	1474.7780	0.0144	0	(14)	38	2		R.FLEQQNQVLQTK.W
269	738.4041	1474.7936	1474.7780	0.0156	0	(11)	74	3		R.FLEQQNQVLQTK.W
270	738.4212	1474.8278	1474.7780	0.0498	0	67	0.00019	1		R.FLEQQNQVLQTK.W

Proteins matching the same set of peptides:
K2C73_MOUSE **Mass:** 58875 **Score:** 75 **Matches:** 4(1) **Sequences:** 2(1)
Keratin, type II cytoskeletal 73 OS=Mus musculus OX=10090 GN=Krt73 PE=1 SV=1

---

10. K2CO_CHICK **Mass:** 53770 **Score:** 72 **Matches:** 2(1) **Sequences:** 2(1) **emPAI:** 0.07
Keratin, type II cytoskeletal cochleal OS=Gallus gallus OX=9031 PE=2 SV=1
☐ Check to include this hit in error tolerant search

Query	Observed	Mr(expt)	Mr(calc)	Delta	Miss	Score	Expect	Rank	Unique	Peptide
233	639.3407	1276.6668	1276.7027	-0.0358	0	9	1.4e+02	4		K.LALDIEIATYR.K
253	665.3858	1328.7570	1328.7187	0.0383	0	63	0.00046	1		R.NLDLDSIIAEVK.A

---

11. K2C7_BOVIN **Mass:** 51546 **Score:** 69 **Matches:** 2(1) **Sequences:** 2(1) **emPAI:** 0.07
Keratin, type II cytoskeletal 7 OS=Bos taurus OX=9913 GN=KRT7 PE=2 SV=1
☐ Check to include this hit in error tolerant search

Query	Observed	Mr(expt)	Mr(calc)	Delta	Miss	Score	Expect	Rank	Unique	Peptide
233	639.3407	1276.6668	1276.7027	-0.0358	0	9	1.4e+02	4		K.LALDIEIATYR.K
249	651.8764	1301.7383	1301.7078	0.0305	0	60	0.001	1		R.SLDLDSIIAEVK.A

---

12. PEX1_HUMAN **Mass:** 142778 **Score:** 44 **Matches:** 3(1) **Sequences:** 1(1) **emPAI:** 0.03
Peroxisome biogenesis factor 1 OS=Homo sapiens OX=9606 GN=PEX1 PE=1 SV=1
☐ Check to include this hit in error tolerant search

	Query	Observed	Mr(expt)	Mr(calc)	Delta	Miss	Score	Expect	Rank	Unique	Peptide
☑	191	533.2566	1064.4986	1064.5358	-0.0372	1	44	0.042	1	U	K.GMMKELQTK.Q
☑	192	533.2695	1064.5244	1064.5358	-0.0114	1	(19)	13	1	U	K.GMMKELQTK.Q
☑	193	533.2699	1064.5252	1064.5358	-0.0106	1	(24)	4.4	1	U	K.GMMKELQTK.Q

Peptide matches not assigned to protein hits: (no details means no match)

	Query	Observed	Mr(expt)	Mr(calc)	Delta	Miss	Score	Expect	Rank	Unique	Peptide
☑	87	420.2303	838.4460	838.3565	0.0895	0	26	1.9	1		SMGIMVEG + Oxidation (M)
☑	189	530.8027	1059.5908	1059.6176	-0.0268	0	24	4.7	1		LTETIGSIVK
☑	155	487.2774	972.5402	972.4698	0.0704	0	22	8.3	1		IENLEPCR
☑	100	428.2297	854.4448	854.4208	0.0241	0	21	6.4	1		TFELAMK + Oxidation (M)
☑	54	400.3000	798.5854	798.5327	0.0527	1	21	5.1	1		KGGVVIVK
☑	178	517.2678	1032.5211	1032.5088	0.0123	0	21	10	1		LTITGNDGDK
☑	77	414.2280	826.4414	826.3717	0.0697	0	20	7.9	1		FPCMVSK + Oxidation (M)
☑	79	414.2395	826.4644	826.3717	0.0927	0	20	8	1		FPCMVSK + Oxidation (M)
☑	71	412.7607	823.5069	823.4262	0.0807	0	18	13	1		VAASMAFK
☑	275	507.2739	1518.8000	1518.6845	0.1155	1	16	22	1		GLENARQEGGEMGR + Oxidation (M)
☑	171	502.2665	1002.5185	1002.5896	-0.0711	1	16	29	1		VGSGVLIKCK
☑	222	414.2314	1239.6723	1239.5884	0.0839	1	16	25	1		ENAESFPKYR
☑	147	472.2926	942.5706	942.5498	0.0207	0	16	30	1		TPTVSALVR
☑	212	400.3000	1197.8782	1197.7445	0.1337	1	16	6.1	1		LLDGSLIAIKR
☑	234	427.2634	1278.7682	1278.6456	0.1226	0	16	24	1		LTEVGVDGFSQK
☑	170	500.2455	998.4765	998.5760	-0.0995	0	16	30	1		ITPVSQLNK
☑	104	428.7817	855.5489	855.5178	0.0311	0	16	26	1		IALSPSIR
☑	166	498.8123	995.6100	995.5036	0.1064	0	15	28	1		EPASGGTLHK
☑	179	517.2799	1032.5453	1032.5088	0.0366	0	15	38	1		LTITGNDGDK
☑	73	413.2000	824.3854	824.3916	-0.0061	0	15	22	1		TYDLEGK
☑	78	414.2391	826.4637	826.3717	0.0920	0	14	30	1		FPCMVSK + Oxidation (M)
☑	209	400.3000	1197.8782	1197.6829	0.1953	1	14	8.8	1		LEAQRLEAIR
☑	295	897.9324	1793.8502	1793.9321	-0.0819	0	14	32	1		CLGHLFLSLGMVYLR + Carbamidomethyl (C); Oxidation
☑	75	414.2174	826.4203	826.5276	-0.1073	0	14	35	1		LIGQVIGK
☑	175	507.3298	1012.6450	1012.6029	0.0421	1	14	40	1		IEAAGGKVIR
☑	146	472.2845	942.5545	942.4593	0.0952	0	13	55	1		MQEPAVPR + Oxidation (M)
	103	428.7813	855.5481	855.4814	0.0667	0	13	45	1		IVDVTPGR

☑										
☑	50	400.2340	798.4534	798.4235	0.0299	0	13	33	1	AIENQPK
☑	196	541.2936	1080.5727	1080.5056	0.0671	0	13	47	1	TLCGALSNMR + Oxidation (M)
☑	169	500.2260	998.4375	998.5621	-0.1246	1	13	54	1	ADALRAAVGR
☑	228	424.2000	1269.5782	1269.5634	0.0147	0	13	51	1	MAAFWDQVMR + Oxidation (M)
☑	53	400.2483	798.4821	798.5076	-0.0254	1	13	35	1	LANVVRK
☑	164	493.3056	984.5967	984.5968	-0.0001	0	13	63	1	ITAVVDVIR
☑	265	480.1871	1437.5394	1437.6592	-0.1198	0	12	37	1	VAAMQGNIEQSMK + 2 Oxidation (M)
☑	76	414.2202	826.4258	826.3974	0.0284	1	12	53	1	RTAADFF
☑	108	432.3000	862.5854	862.4946	0.0909	1	12	71	1	MALTAKTK
☑	180	520.2300	1038.4455	1038.4903	-0.0448	0	12	63	1	MDTTTVLDK + Oxidation (M)
☑	211	400.3000	1197.8782	1197.6829	0.1953	1	12	15	1	AGAAAAASQKKPK
☑	121	436.1877	870.3608	870.5287	-0.1679	0	12	75	1	QASLAIIR
☑	56	400.3000	798.5854	798.4851	0.1004	0	11	43	1	LVEIPTK
☑	214	402.3000	1203.8782	1203.7339	0.1443	1	11	11	1	GYKITVAAILR
☑	80	414.2423	826.4701	826.3201	0.1500	0	11	73	1	MESMDAK + Oxidation (M)
☑	110	432.3000	862.5854	862.4946	0.0909	1	11	98	1	MTLLKNK + Oxidation (M)
☑	207	592.8297	1183.6449	1183.6561	-0.0112	0	11	91	1	NVEIAVQINGK
☑	250	436.9180	1307.7322	1307.7310	0.0013	1	11	87	1	IEDGHVRLLTR
☑	183	523.2802	1044.5459	1044.5815	-0.0356	0	10	1.2e+02	1	LSQITQDIK
☑	83	416.2486	830.4827	830.4974	-0.0146	1	10	1.2e+02	1	ISVTQKR
☑	136	449.2974	896.5802	896.4756	0.1045	0	10	74	1	YGFVGGLGK
☑	68	406.2000	810.3854	810.5075	-0.1221	1	10	52	1	NPIAKIR
☑	109	432.3000	862.5854	862.5025	0.0830	1	10	1.1e+02	1	GYLIKNR
☑	285	421.2404	1680.9323	1680.7640	0.1683	0	10	82	1	SAFQSSICSGFIGFCK
☑	123	437.2578	872.5010	872.5080	-0.0070	0	10	1.4e+02	1	VAIVTGASR
☑	142	457.2719	912.5292	912.4552	0.0739	0	10	1.1e+02	1	QLSYTSSK
☑	216	402.3000	1203.8782	1203.7261	0.1521	1	10	14	1	MTILKQLITK + Oxidation (M)
☑	176	514.3000	1026.5854	1026.5822	0.0033	0	10	1.1e+02	1	NLPSTQVLR
☑	165	498.8119	995.6092	995.5400	0.0692	1	10	1e+02	1	IPSTEGKHK
☑	154	486.3320	970.6494	970.5633	0.0861	0	10	1.1e+02	1	KPNQMLIK
☑	55	400.3000	798.5854	798.4460	0.1394	0	10	66	1	INSGRPR
☑	210	400.3000	1197.8782	1197.6829	0.1953	1	10	25	1	AGAAAAASQKKPK
☑	274	505.3000	1512.8782	1512.7680	0.1102	0	10	96	1	LLMIIIDSYMNR + 2 Oxidation (M)
☑	255	446.2170	1335.6291	1335.6526	-0.0235	1	9	1.2e+02	1	DMLGGLMPKNTK + 2 Oxidation (M)
☑	257	681.2785	1360.5425	1360.5097	0.0328	0	9	1e+02	1	EYTMQGCPECK + Carbamidomethyl (C); Oxidation (M)
☑	158	488.2734	974.5323	974.4417	0.0906	0	9	1.7e+02	1	VNGEGGGGNSK
☑	201	568.3403	1134.6660	1134.7012	-0.0352	0	9	1.5e+02	1	TPVATPVLIPK
☑	63	402.3000	802.5854	802.4371	0.1484	0	9	1.2e+02	1	LIDACLR
☑	157	487.2874	972.5602	972.5392	0.0210	1	9	1.8e+02	1	IEIEKWR
☑	59	400.3546	798.6946	798.4963	0.1983	0	9	28	1	IIDGLLR
☑	273	498.9394	1493.7964	1493.6681	0.1283	1	9	1.3e+02	1	DQRWNCSSLETR
☑	111	432.3000	862.5854	862.4582	0.1272	1	8	1.6e+02	1	KMEAIQK + Oxidation (M)
☑	163	493.2425	984.4704	984.4620	0.0084	1	8	1.7e+02	1	TMTEKMTK + Oxidation (M)
☑	122	437.2435	872.4724	872.4504	0.0219	0	8	2e+02	1	QAPASPFR
☑	58	400.3523	798.6900	798.4963	0.1936	0	8	30	1	IDIALVR
☑	101	428.2468	854.4790	854.5086	-0.0296	1	8	1.2e+02	1	LVGRASPR
☑	174	505.3000	1008.5854	1008.6008	-0.0154	0	8	1.6e+02	1	ILIFFQTK
☑	236	640.8000	1279.5854	1279.6516	-0.0661	1	8	1.6e+02	1	KMLVDTIEGMK + Oxidation (M)
☑	72	412.7699	823.5252	823.4222	0.1030	1	8	1.1e+02	1	VMSRTSK + Oxidation (M)
☑	140	450.2911	898.5677	898.4218	0.1458	0	8	1.6e+02	1	GYMVAAGSK + Oxidation (M)
☑	172	504.2374	1006.4603	1006.4614	-0.0011	1	8	1.9e+02	1	QSRMENSR
☑	181	521.2606	1040.5066	1040.4314	0.0752	1	8	1.7e+02	1	RMAMSCGGR + Carbamidomethyl (C); Oxidation (M)
☑	276	514.3000	1539.8782	1539.7762	0.1020	1	8	1.4e+02	1	MSNHRILHTMLR + 2 Oxidation (M)
☑	102	428.2700	854.5255	854.3882	0.1373	0	8	1.3e+02	1	NEDVHNK
☑	187	530.2997	1058.5849	1058.5356	0.0492	0	8	2.1e+02	1	LTNIQQQSQ
☑	120	436.1410	870.2674	870.4195	-0.1521	0	8	70	1	NDASSLHK
☑	218	404.3000	1209.8782	1209.7016	0.1766	1	8	28	1	MKLAVLAVGHR + Oxidation (M)
☑	221	413.2000	1236.5782	1236.5921	-0.0139	1	8	1.7e+02	1	YTGVAPRDQCK
☑	204	585.3853	1168.7561	1168.6815	0.0745	0	7	1.4e+02	1	EAALVVEGLLR
☑	74	413.2359	824.4572	824.4504	0.0067	0	7	1.2e+02	1	QSPPLAGR
☑	96	427.2749	852.5353	852.3899	0.1454	0	7	1.4e+02	1	MEVTSSLA + Oxidation (M)
☑	57	400.3000	798.5854	798.4422	0.1433	0	7	1.1e+02	1	IMSAHIK
☑	143	462.1924	922.3702	922.4396	-0.0693	0	7	2.1e+02	1	LEEGNSFK
☑	153	485.3276	968.6407	968.4749	0.1658	0	7	1.5e+02	1	TPEGHGMLK
☑	62	402.3000	802.5854	802.4548	0.1306	0	7	1.8e+02	1	LLDSSLR
☑	252	441.7993	1322.3762	1322.5383	-0.1621	0	7	14	1	ENHQMTCPYK + Carbamidomethyl (C); Oxidation (M)
☑	112	432.3000	862.5854	862.4218	0.1636	1	7	2.4e+02	1	LREMVEA + Oxidation (M)
☑	131	448.2576	894.5007	894.3688	0.1320	0	7	2.1e+02	1	MASCNPTR + Oxidation (M)
☑	88	420.2604	838.5062	838.3565	0.1498	0	7	1.5e+02	1	SMGIMVEG + Oxidation (M)
☑	303	990.3715	1978.7285	1978.9063	-0.1778	0	7	44	1	MVSSVLEVSHVFCCPNR + Carbamidomethyl (C); Oxidatio
☑	186	525.8539	1049.6932	1049.5063	0.1870	0	6	2.1e+02	1	DLAAMGESLK + Oxidation (M)
☑	52	400.2444	798.4743	798.4058	0.0685	0	6	1.6e+02	1	MHLPASK + Oxidation (M)
☑	116	434.7837	867.5528	867.4562	0.0965	1	6	2e+02	1	REDAIHK
☑	213	401.5000	1201.4782	1201.6568	-0.1786	1	6	2.4e+02	1	ATGKGYLHVTR
☑	161	492.2962	982.5779	982.4654	0.1124	0	6	2.4e+02	1	NNPLCTHK + Carbamidomethyl (C)
☑	287	430.1484	1716.5647	1716.6299	-0.0652	1	6	23	1	CCMSHCTDFCRNPGK + Oxidation (M)
☑	139	450.2905	898.5664	898.4436	0.1228	0	6	2.7e+02	1	FSIFEEK
☑	219	406.2000	1215.5782	1215.6686	-0.0904	0	6	2.9e+02	1	QPAVPAVFLMK + Oxidation (M)
☑	65	404.3000	806.5854	806.4134	0.1721	1	6	1.5e+02	1	KTGASSEK
☑	144	462.2518	922.4891	922.5236	-0.0345	0	6	3.1e+02	1	GDPKPGKPK
☑	316	585.3048	2337.1900	2337.1708	0.0192	1	6	1.9e+02	1	AAAKLVFMASQAGGMEIEEVAAK + Oxidation (M)
☑	86	420.1994	838.3843	838.4041	-0.0198	1	6	2e+02	1	GMGVKGMK + 2 Oxidation (M)
☑	225	419.3189	1254.9350	1254.7660	0.1691	1	5	23	1	VVTDALGALRLK
☑	91	424.2000	846.3854	846.4083	-0.0228	0	5	3.4e+02	1	TEAEAQAK
	61	402.3000	802.5854	802.4337	0.1517	0	5	2.7e+02	1	YVHVGTK

☑										
☑	118	435.2764	868.5382	868.4225	0.1157	0	5	2.5e+02	1	HMIHTSK + Oxidation (M)
☑	117	434.7919	867.5692	867.4160	0.1532	0	5	2.6e+02	1	MNTVPYK + Oxidation (M)
☑	282	553.6000	1657.7782	1657.8709	-0.0927	0	5	3e+02	1	ILQEAVAVGVGVMDNK + Oxidation (M)
☑	280	818.3000	1634.5854	1634.7149	-0.1295	0	5	94	1	VHQMINNINMMCR + 2 Oxidation (M)
☑	97	427.3048	852.5950	852.5181	0.0769	0	5	2e+02	1	AGVPAAVIR
☑	264	704.2357	1406.4568	1406.6435	-0.1866	0	5	67	1	ASLACSSWPLCR + 2 Carbamidomethyl (C)
☑	105	431.1000	860.1854	860.3777	-0.1922	0	5	23	1	WGDVNDR
☑	94	426.2873	850.5601	850.4041	0.1561	0	5	3.2e+02	1	EMVLMGR + Oxidation (M)
☑	145	464.2758	926.5371	926.4821	0.0550	0	5	3.6e+02	1	GPNGLDINK
☑	243	432.3000	1293.8782	1293.7194	0.1588	1	4	1.2e+02	1	VFSKPRFGQTK
☑	106	432.1884	862.3623	862.4661	-0.1038	0	4	4.6e+02	1	ATAAFNIR
☑	51	400.2415	798.4685	798.4170	0.0515	1	4	2.7e+02	1	ICKHGGK + Carbamidomethyl (C)
☑	134	449.2385	896.4625	896.4208	0.0417	1	4	3.4e+02	1	MTMRGASK + Oxidation (M)
☑	173	504.2425	1006.4704	1006.4832	-0.0128	1	4	4.9e+02	1	KSPFDSGNR
☑	168	498.8145	995.6144	995.5036	0.1108	0	4	3.8e+02	1	NSPQASPPAK
☑	254	445.1393	1332.3959	1332.5728	-0.1769	0	4	39	1	AAAEGGCGAGVEADR
☑	223	417.2000	1248.5782	1248.6206	-0.0424	0	4	4.6e+02	1	SLEMPVIMASR + Oxidation (M)
☑	310	702.2326	2103.6761	2103.8507	-0.1747	0	4	9.2	1	TMQTSQDSMPSMSLNDSGR + 2 Oxidation (M)
☑	244	432.3000	1293.8782	1293.6785	0.1997	1	3	1.5e+02	1	KMVNILMANTK + 2 Oxidation (M)
☑	245	432.3000	1293.8782	1293.6864	0.1918	1	3	1.5e+02	1	TFTGMPRLTVR + Oxidation (M)
☑	182	523.1415	1044.2685	1044.4593	-0.1908	1	3	46	1	NKHGCDICR
☑	251	441.7954	1322.3644	1322.5408	-0.1765	1	3	24	1	EDDAQCQGKDSK
☑	107	432.3000	862.5854	862.4946	0.0909	1	3	5.4e+02	1	MALTAKTK
☑	306	999.7910	1997.5675	1997.7376	-0.1701	0	3	2.2	1	GQCCMPVDGDTYTYGCGR + Carbamidomethyl (C); Oxidati
☑	298	463.1590	1848.6067	1848.7295	-0.1227	0	3	28	1	GLCNFLMSDEDYDDR + Carbamidomethyl (C)
☑	283	555.6000	1663.7782	1663.7334	0.0448	1	3	4.1e+02	1	CSSCGEGFANTYGLKK
☑	238	430.1498	1287.4276	1287.6169	-0.1893	0	3	1.5e+02	1	YTCASIFAEVGK
☑	137	449.3480	896.6814	896.5444	0.1371	0	3	1.6e+02	1	VPVVASVAR
☑	215	402.3000	1203.8782	1203.7088	0.1694	0	3	71	1	ALGGAIIHPSIR
☑	239	431.1000	1290.2782	1290.4461	-0.1680	0	3	1.7	1	TGNCTCGGSCTCK + Carbamidomethyl (C)
☑	167	498.8137	995.6128	995.5988	0.0140	1	3	5.1e+02	1	RELVRPAR
☑	126	441.8023	881.5900	881.4025	0.1875	1	3	4.8e+02	1	NSRMGSSK + Oxidation (M)
☑	300	628.2320	1881.6741	1881.7864	-0.1123	1	2	91	1	YGDEDEIEQEQEDRK
☑	138	450.2858	898.5571	898.4218	0.1353	0	2	5.9e+02	1	GYMVAAGSK + Oxidation (M)
☑	261	463.1571	1386.4494	1386.6449	-0.1955	0	2	1.1e+02	1	MSGGYTSLESSIR
☑	92	425.2699	848.5252	848.4239	0.1012	1	2	7.2e+02	1	TEAEGSKK
☑	232	425.7861	1274.3364	1274.4842	-0.1478	1	2	25	1	GCKCLSNGYCG + 3 Carbamidomethyl (C)
☑	141	455.2641	908.5137	908.5153	-0.0016	1	2	6.6e+02	1	IKLMFNK + Oxidation (M)
☑	150	480.1863	958.3581	958.4654	-0.1073	0	2	7.2e+02	1	MAAGAPAAGAR + Oxidation (M)
☑	242	432.3000	1293.8782	1293.6864	0.1918	1	2	2.1e+02	1	TFTGMPRLTVR + Oxidation (M)
☑	311	703.2455	2106.7147	2106.9139	-0.1992	0	2	31	1	GMNDCLPEQSGLWQWAEK + Oxidation (M)
☑	84	417.2000	832.3854	832.4331	-0.0476	0	2	7.7e+02	1	YTPPEVK
☑	149	477.3294	952.6443	952.4462	0.1982	1	2	5.4e+02	1	SDGTSKSGSK
☑	148	477.2887	952.5629	952.4800	0.0829	0	2	7.1e+02	1	TYGICGAIR
☑	70	818.3000	817.2927	817.3970	-0.1043	0	2	8.9e+02	1	GDEIAWK
☑	309	523.1466	2088.5575	2088.7347	-0.1772	0	1	1	1	DCEDGSDEEGCSCPPNTFK + Carbamidomethyl (C)
☑	115	434.2701	866.5257	866.5086	0.0171	1	1	6.7e+02	1	HVNTLKR
☑	127	441.8037	881.5928	881.4971	0.0958	0	1	6.7e+02	1	AATQPAPVK
☑	320	927.4760	2779.4063	2779.4297	-0.0235	0	1	4.2e+02	1	VALVSFVMVVIGFLMKPCLNHMDGK + 2 Oxidation (M)
☑	293	445.1405	1776.5331	1776.7276	-0.1946	1	1	21	1	CTACPQGCLQCSHRDR
☑	128	447.3608	892.7071	892.5382	0.1690	1	0	1.1e+02	1	LQKYLTK
☑	226	419.3315	1254.9727	1254.7772	0.1955	1	0	12	1	LARLSSGVALLR
☑	241	431.1135	1290.3188	1290.5090	-0.1902	1	0	15	1	ACMHDRCQCPK
☑	229	424.3184	1269.9334	1269.7553	0.1781	0	0	97	1	CVVMGIPLVIVK
☑	129	447.3630	892.7114	892.5130	0.1984	0	0	1.2e+02	1	APQQPKPK
☑	1	*400.2701*	*399.2628*							
☑	2	*400.3000*	*399.2927*							
☑	3	*400.3000*	*399.2927*							
☑	4	*400.3000*	*399.2927*							
☑	5	*400.3000*	*399.2927*							
☑	6	*401.2000*	*400.1927*							
☑	7	*401.5000*	*400.4927*							
☑	8	*402.3000*	*401.2927*							
☑	9	*402.3000*	*401.2927*							
☑	10	*402.3000*	*401.2927*							
☑	11	*402.4000*	*401.3927*							
☑	12	*403.2085*	*402.2013*							
☑	13	*403.2672*	*402.2600*							
☑	14	*404.2116*	*403.2043*							
☑	15	*404.3000*	*403.2927*							
☑	16	*406.2000*	*405.1927*							
☑	17	*406.4000*	*405.3927*							
☑	18	*413.2000*	*412.1927*							
☑	19	*417.2000*	*416.1927*							
☑	20	*417.4000*	*416.3927*							
☑	21	*421.2535*	*420.2462*							
☑	22	*424.2000*	*423.1927*							
☑	23	*425.3268*	*424.3195*							
☑	24	*427.3997*	*426.3924*							
☑	25	*427.3997*	*426.3925*							
☑	26	*431.1000*	*430.0927*							
☑	27	*432.3000*	*431.2927*							
☑	28	*432.3000*	*431.2927*							
☑	29	*432.3000*	*431.2927*							
	30	*432.3000*	*431.2927*							

☑			
☑	31	432.3000	431.2927
☑	32	432.4000	431.3927
☑	33	432.4000	431.3927
☑	34	434.2735	433.2662
☑	35	436.2261	435.2188
☑	36	439.3833	438.3760
☑	37	444.2171	443.2098
☑	38	447.2128	446.2055
☑	39	447.2235	446.2162
☑	40	456.3204	455.3131
☑	41	461.3988	460.3915
☑	42	474.2418	473.2345
☑	43	484.4113	483.4040
☑	44	497.4203	496.4130
☑	45	505.3000	504.2927
☑	46	514.3000	513.2927
☑	47	553.6000	552.5927
☑	48	555.6000	554.5927
☑	49	640.8000	639.7927
☑	60	401.5000	800.9854
☑	64	402.4000	802.7854
☑	66	404.3577	806.7009
☑	67	405.3607	808.7067
☑	69	406.4000	810.7854
☑	81	414.3249	826.6353
☑	82	414.3365	826.6585
☑	85	417.4000	832.7854
☑	93	425.3822	848.7499
☑	95	427.2583	852.5020
☑	98	427.3922	852.7699
☑	99	427.4029	852.7912
☑	113	432.4000	862.7854
☑	114	432.4000	862.7854
☑	119	435.3790	868.7435
☑	130	447.3643	892.7140
☑	151	481.4476	960.8806
☑	159	491.3412	980.6678
☑	160	491.4320	980.8494
☑	162	492.2998	982.5850
☑	185	525.4447	1048.8749
☑	198	553.6000	1105.1854
☑	199	555.6000	1109.1854
☑	217	402.4000	1204.1782
☑	220	406.4000	1216.1782
☑	224	417.4000	1249.1782
☑	230	424.3284	1269.9634
☑	231	425.3839	1273.1299
☑	235	427.4030	1279.1871
☑	237	428.7784	1283.3132
☑	240	431.1114	1290.3125
☑	246	432.3000	1293.8782
☑	247	432.4000	1294.1782
☑	248	432.4000	1294.1782
☑	266	482.0428	1443.1066
☑	271	498.3782	1492.1128
☑	277	523.1512	1566.4318
☑	286	570.3813	1708.1220
☑	288	431.1177	1720.4419
☑	289	436.5673	1742.2403
☑	292	440.5604	1758.2123
☑	302	640.8000	1919.3782
☑	305	499.3450	1993.3508
☑	307	523.1340	2088.5067
☑	308	523.1348	2088.5102
☑	319	818.3000	2451.8782
☑	321	702.2385	2804.9250

Search Parameters

```
Type of search         : MS/MS Ion Search
Enzyme                 : Trypsin
Variable modifications : Carbamidomethyl (C),Oxidation (M)
Mass values            : Monoisotopic
Protein Mass           : Unrestricted
Peptide Mass Tolerance : ± 0.2 Da
Fragment Mass Tolerance: ± 0.2 Da
Max Missed Cleavages   : 1
Instrument type        : Default
Number of queries      : 323
```
