# Supplementary material for: Neuron-associated retroelement-derived protein Arc/Arg3.1 assists in the early stages of alphaherpesvirus infection in human neuronal cells
Source: PLoS One. 2024 Dec 12;19(12):e0314980. doi: 10.1371/journal.pone.0314980 (PMC11637343; doi:10.1371/journal.pone.0314980)
Supplement: S1 Raw images — (PDF) [file pone.0314980.s003.pdf]

Fig. 1A

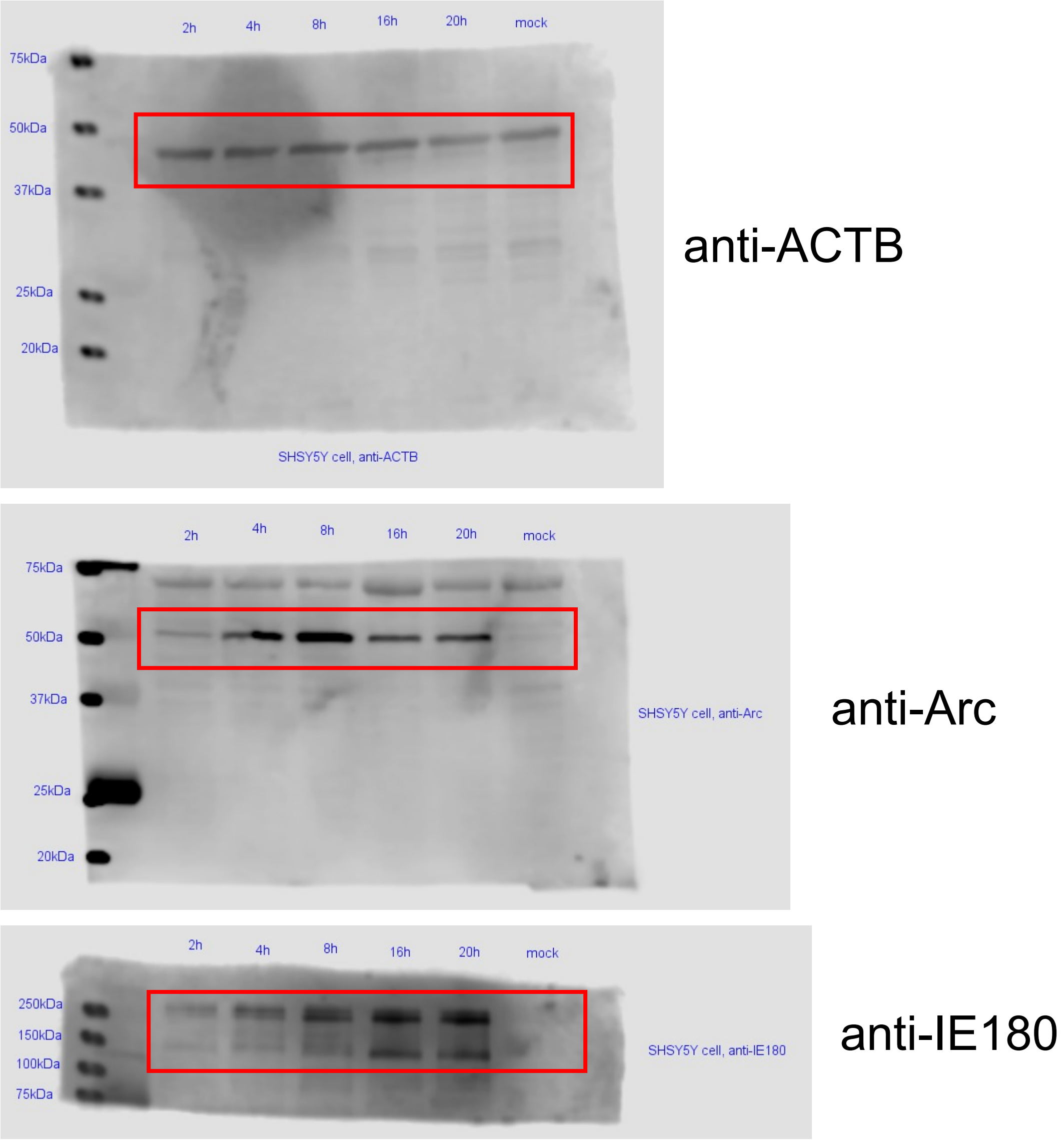
2h
4h
8h
16h
20h
mock
75kDa
50kDa
37kDa
25kDa
20kDa
SHSY5Y cell, anti-ACTB
anti-ACTB
2h
4h
8h
16h
20h
mock
75kDa
50kDa
37kDa
25kDa
20kDa
SHSY5Y cell, anti-Arc
anti-Arc
2h
4h
8h
16h
20h
mock
250kDa
150kDa
100kDa
75kDa
SHSY5Y cell, anti-IE180
anti-IE180

Fig. 1C

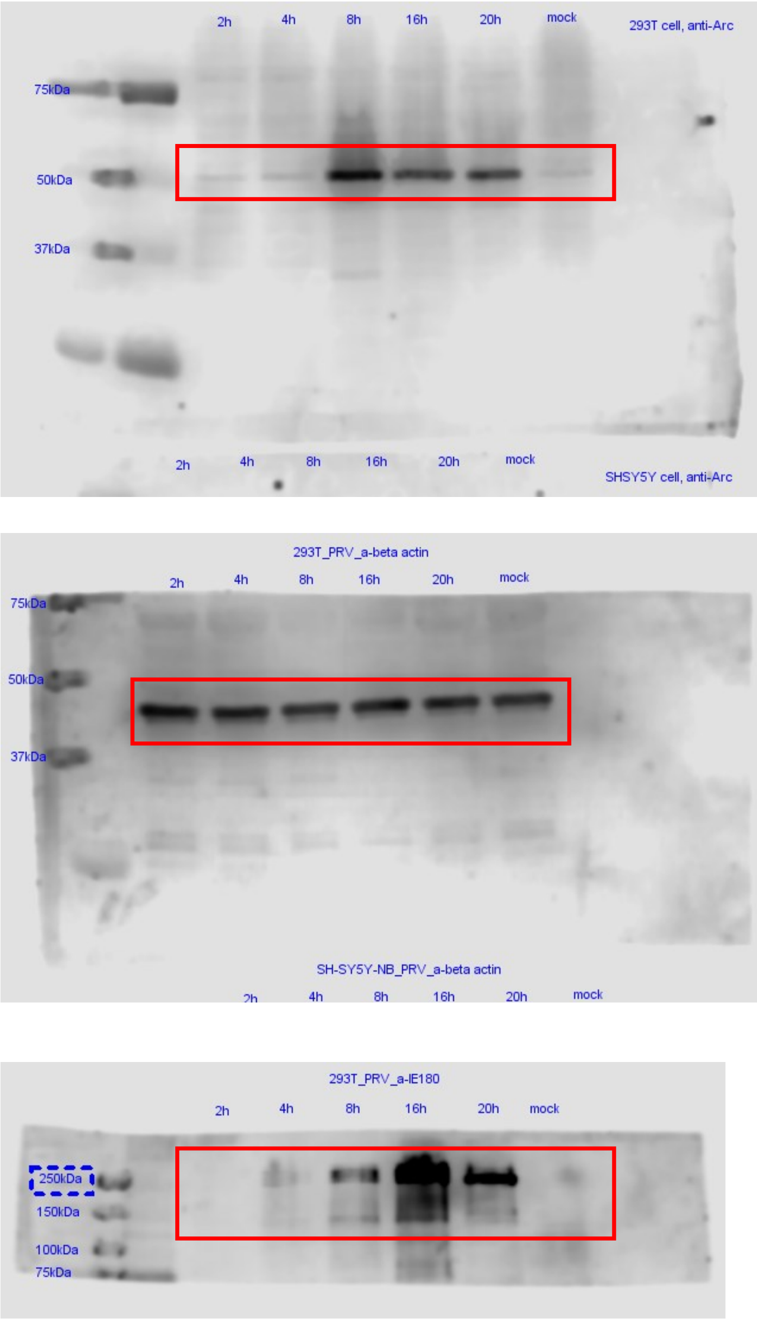

anti-Arc

anti-ACTB

anti-IE180

Fig. 1E

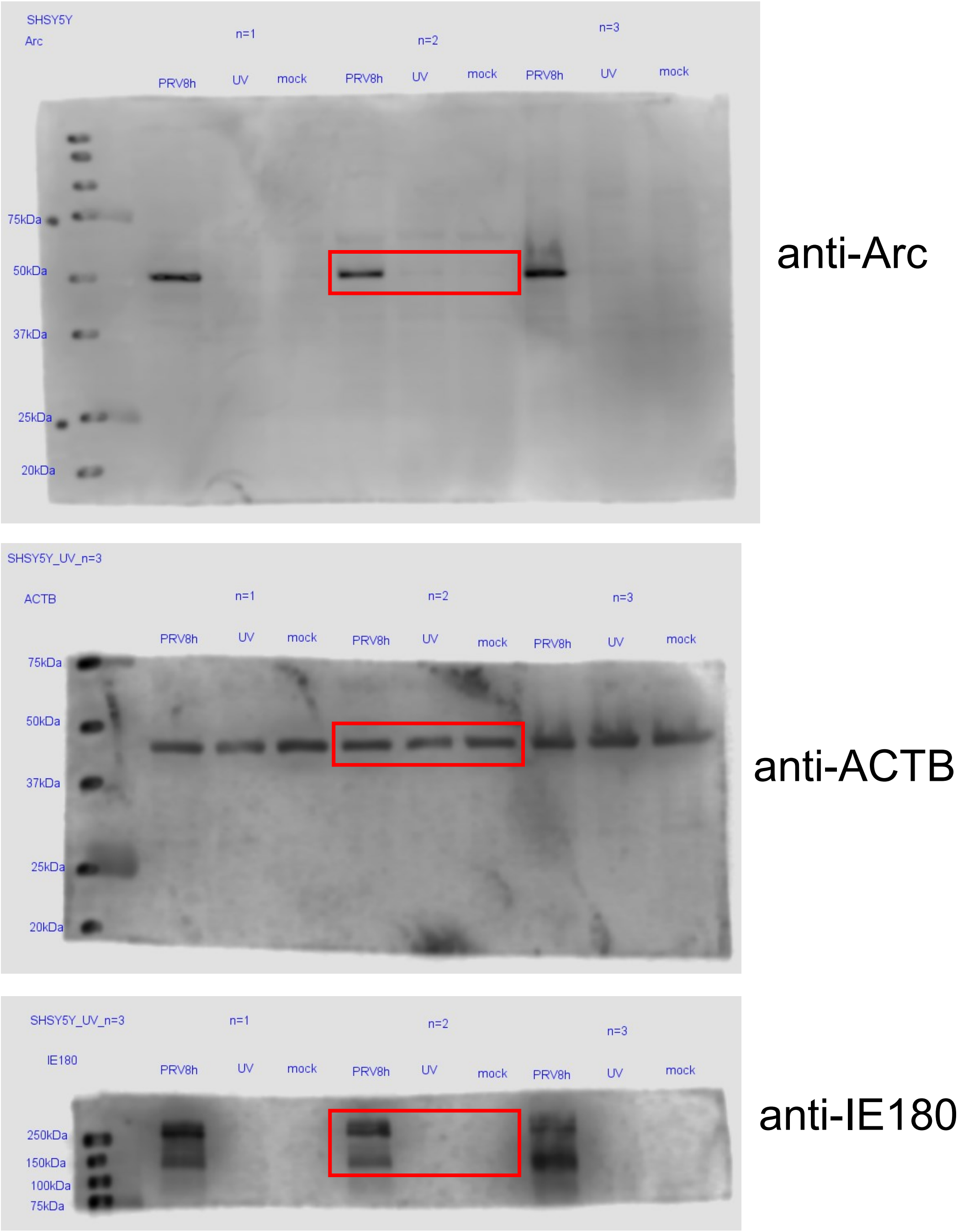
SHSY5Y
Arc
n=1
n=2
n=3
PRV8h
UV
mock
PRV8h
UV
mock
PRV8h
UV
mock
75kDa
50kDa
37kDa
25kDa
20kDa
anti-Arc
SHSY5Y_UV_n=3
ACTB
n=1
n=2
n=3
PRV8h
UV
mock
PRV8h
UV
mock
PRV8h
UV
mock
75kDa
50kDa
37kDa
25kDa
20kDa
anti-ACTB
SHSY5Y_UV_n=3
IE180
n=1
n=2
n=3
PRV8h
UV
mock
PRV8h
UV
mock
PRV8h
UV
mock
250kDa
150kDa
100kDa
75kDa
anti-IE180

Fig. 1G

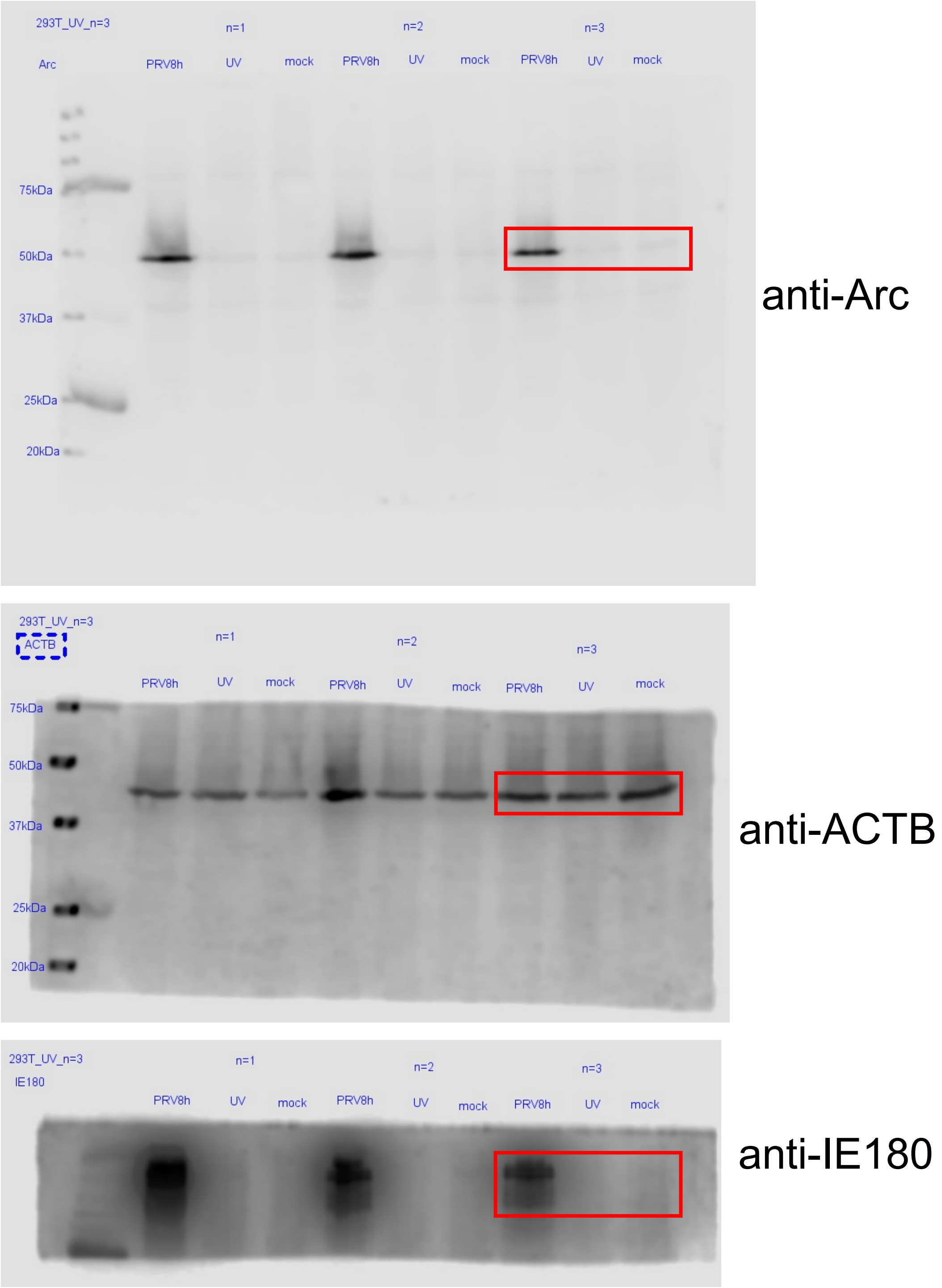

293T_UV_n=3
Arc
n=1
n=2
n=3
PRV8h
UV
mock
PRV8h
UV
mock
PRV8h
UV
mock
75kDa
50kDa
37kDa
25kDa
20kDa
anti-Arc
293T_UV_n=3
ACTB
n=1
n=2
n=3
PRV8h
UV
mock
PRV8h
UV
mock
PRV8h
UV
mock
75kDa
50kDa
37kDa
25kDa
20kDa
anti-ACTB
293T_UV_n=3
IE180
n=1
n=2
n=3
PRV8h
UV
mock
PRV8h
UV
mock
PRV8h
UV
mock
anti-IE180

Fig. 2A

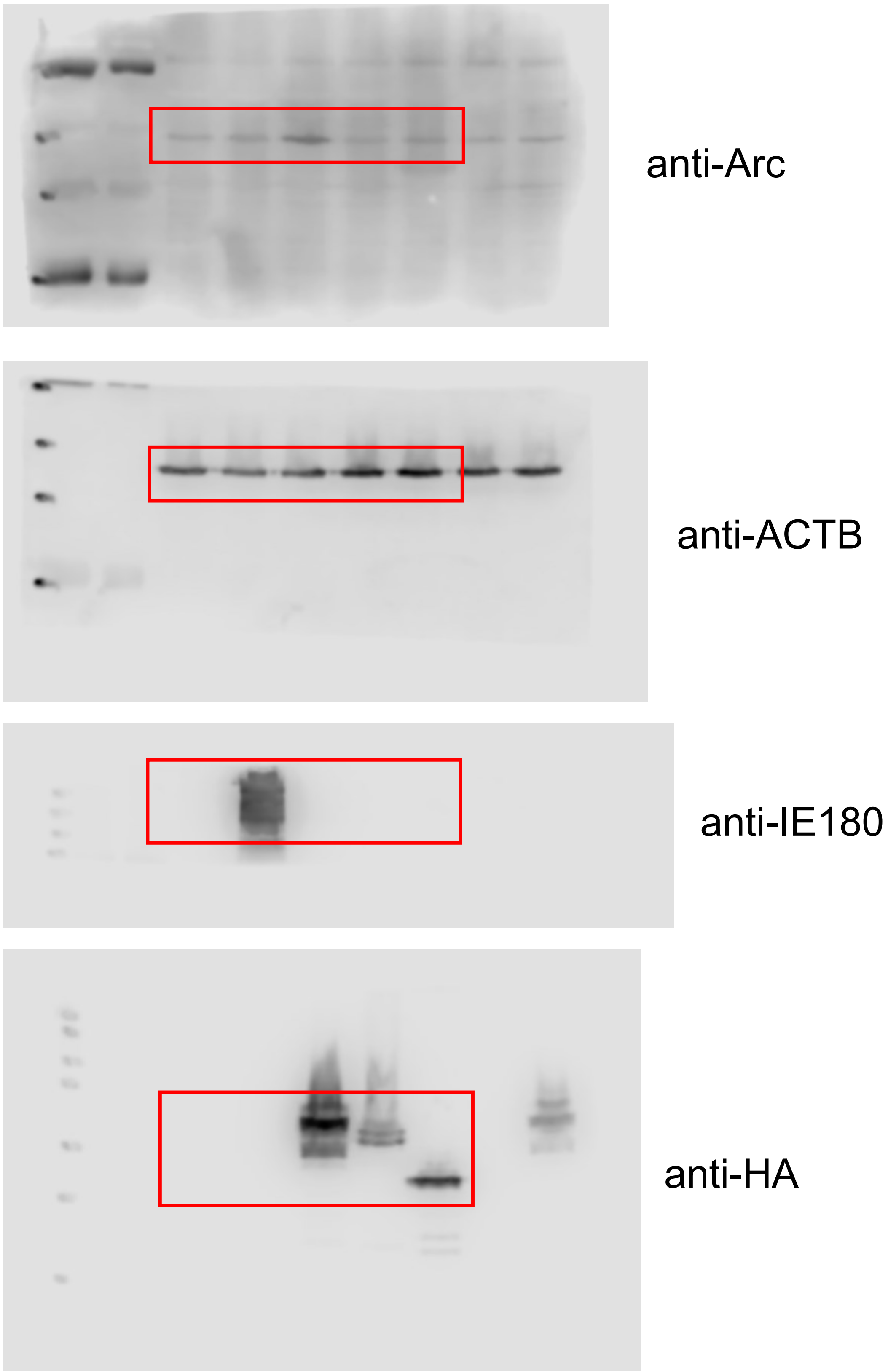

Fig. 2B

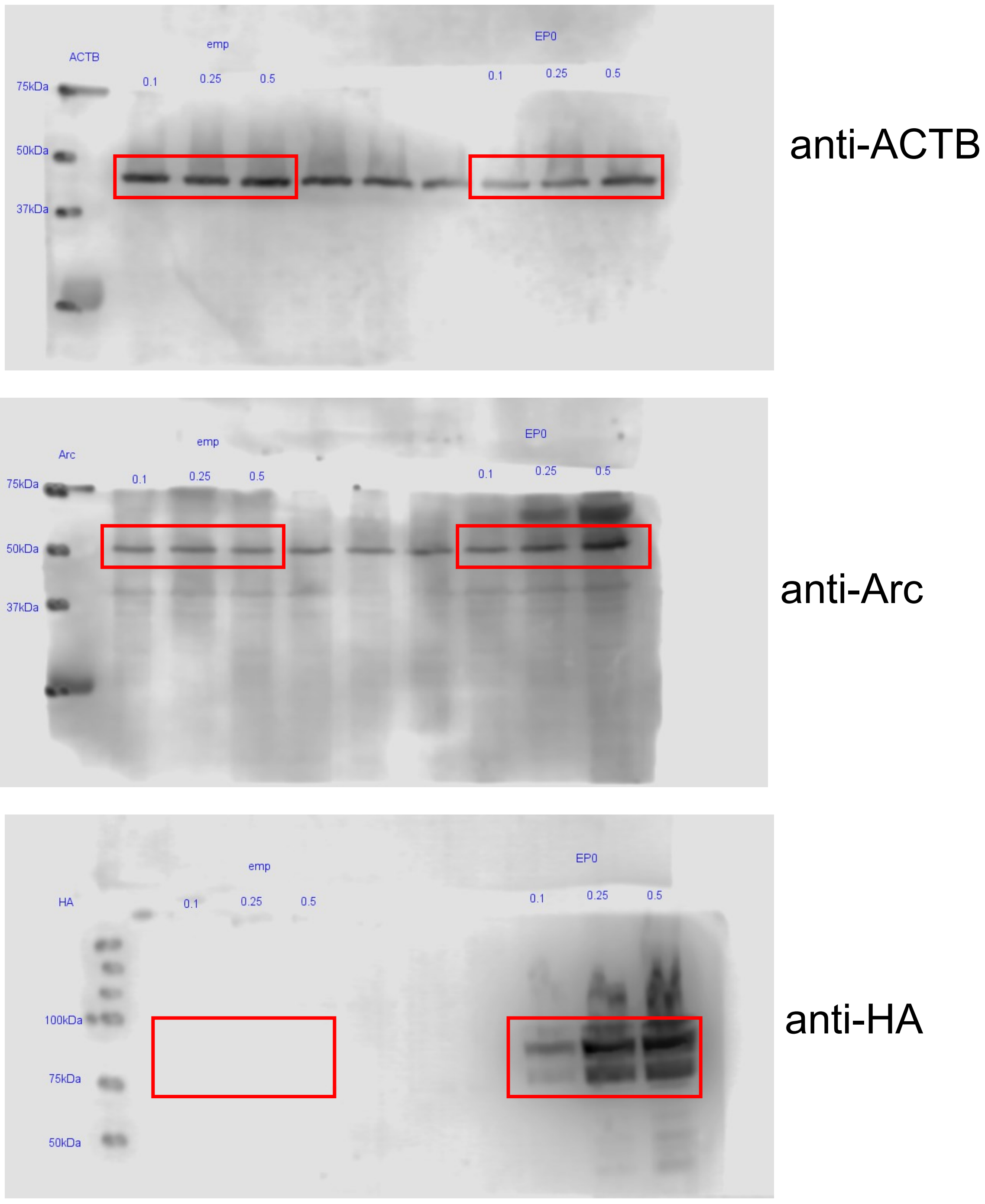
ACTB
emp
EP0
75kDa
50kDa
37kDa
0.1
0.25
0.5
0.1
0.25
0.5
anti-ACTB
Arc
emp
EP0
75kDa
50kDa
37kDa
0.1
0.25
0.5
0.1
0.25
0.5
anti-Arc
HA
emp
EP0
0.1
0.25
0.5
0.1
0.25
0.5
100kDa
75kDa
50kDa
anti-HA

Fig. 2D

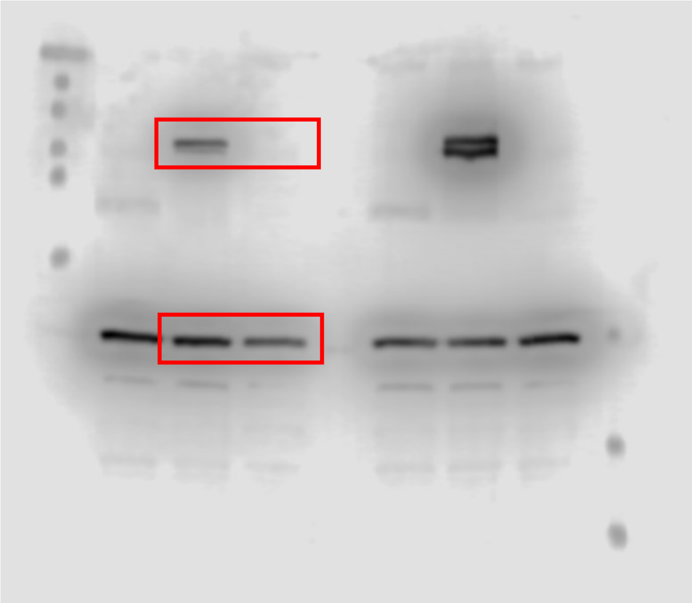

anti-HA

anti-GAPDH

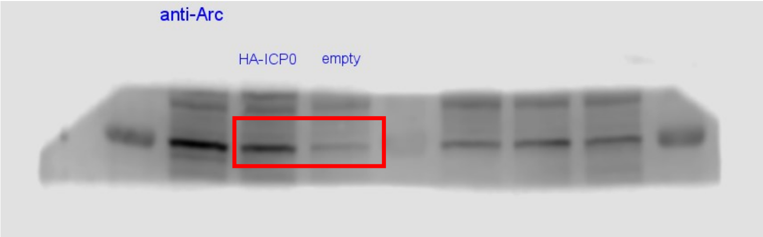

anti-Arc

Fig. 3A

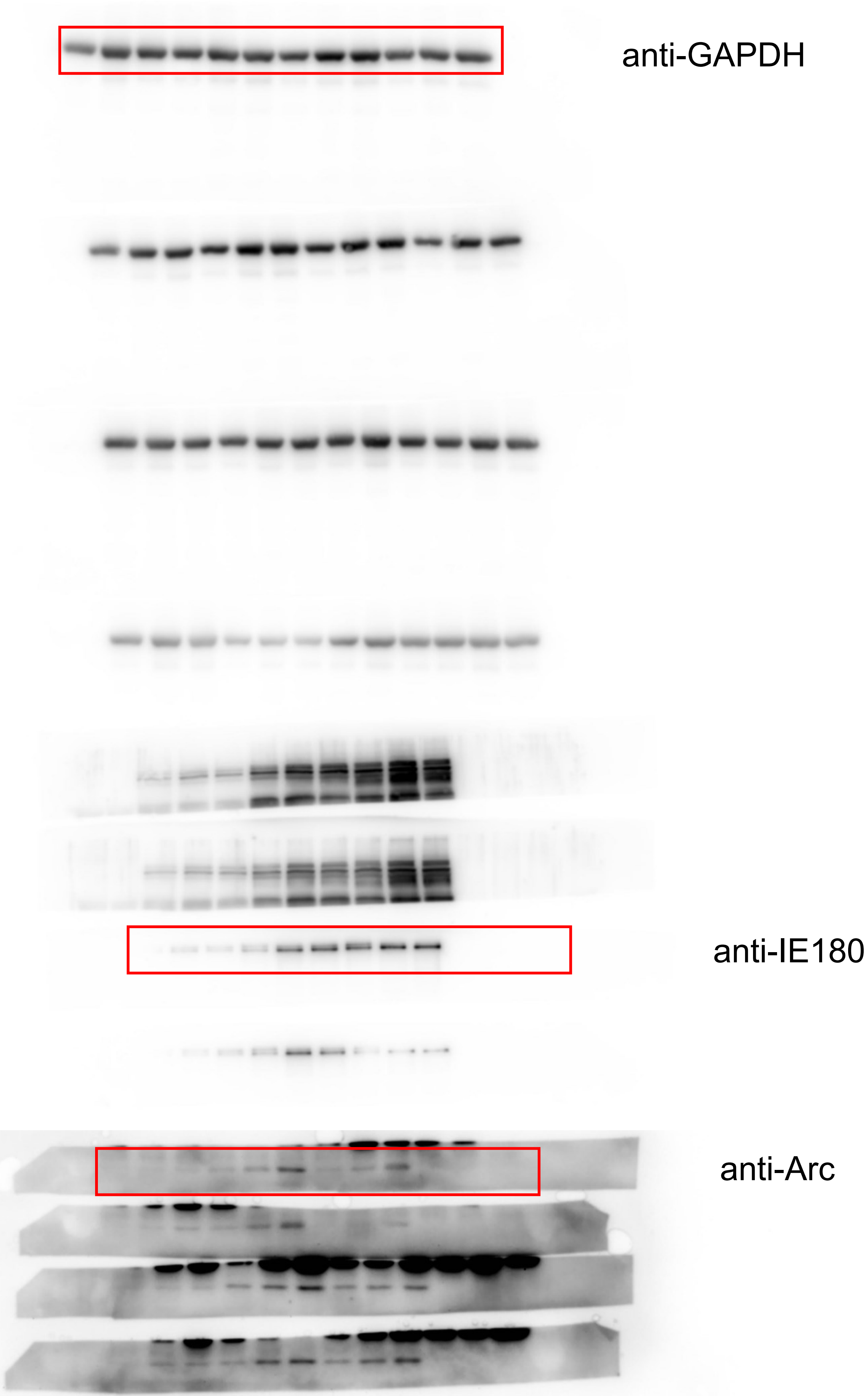

Fig. 3B

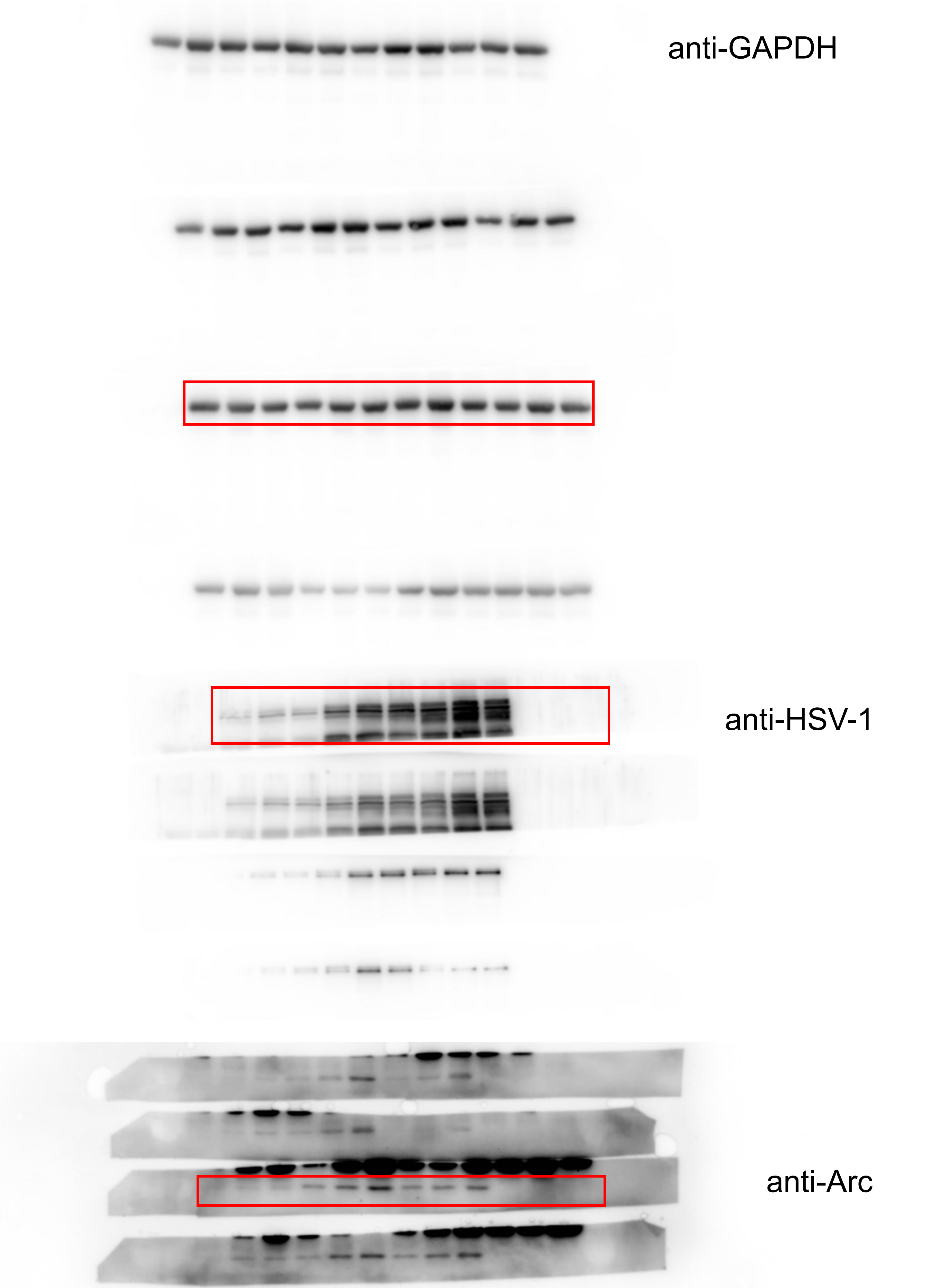

Fig. 4A

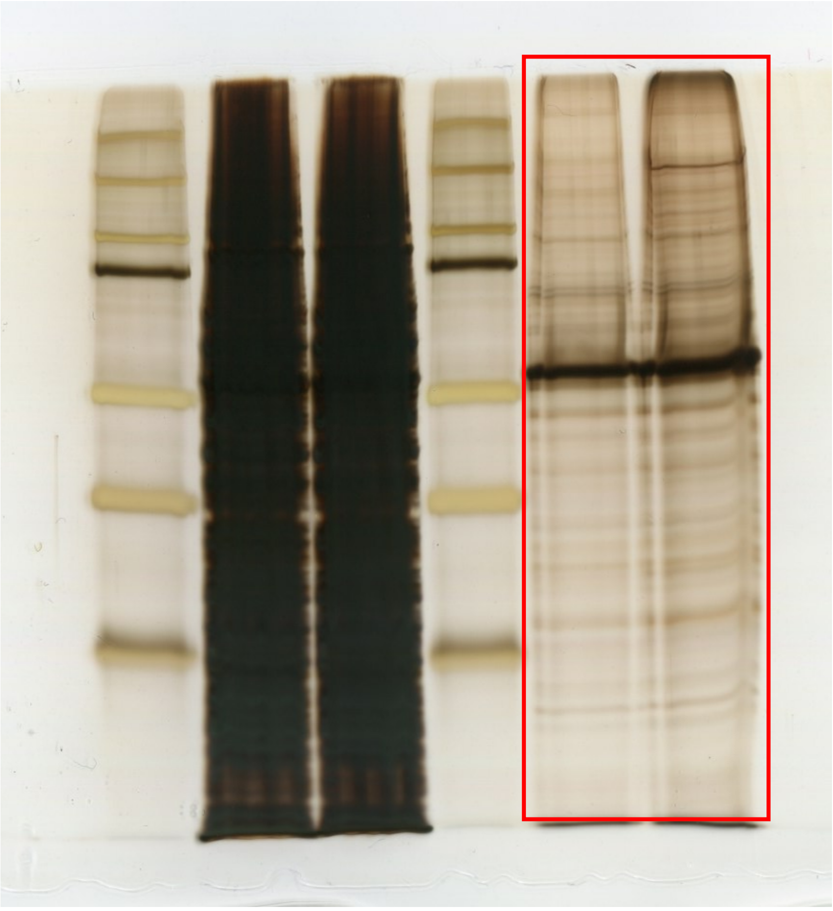

Fig. 4B

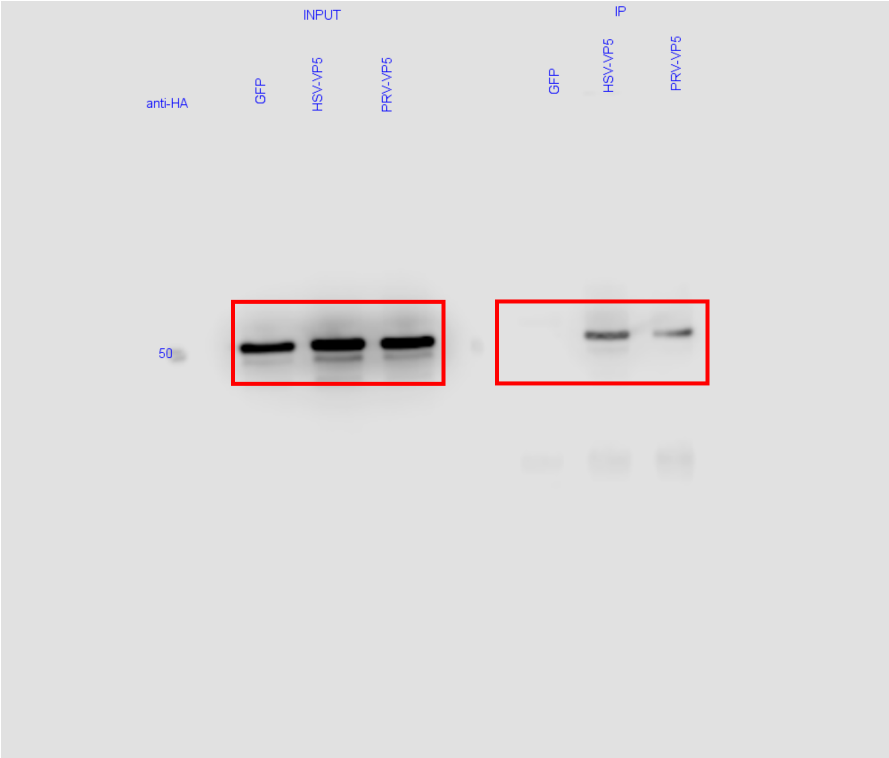

anti-HA (Arc)

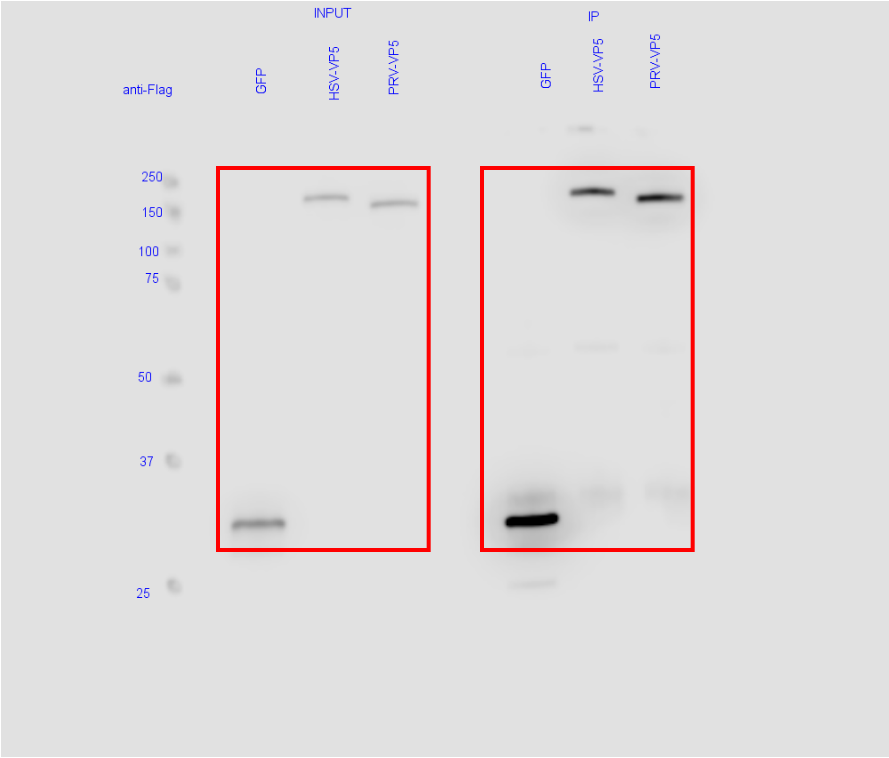

anti-Flag
(VP5 and GFP)

Fig. 4B

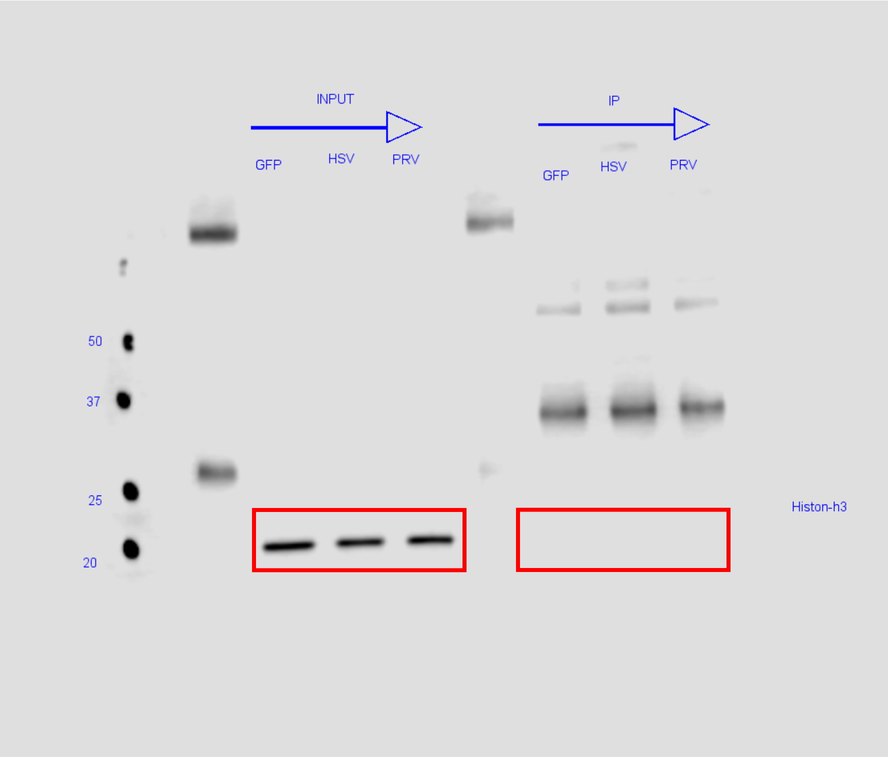

anti-Histone-H3

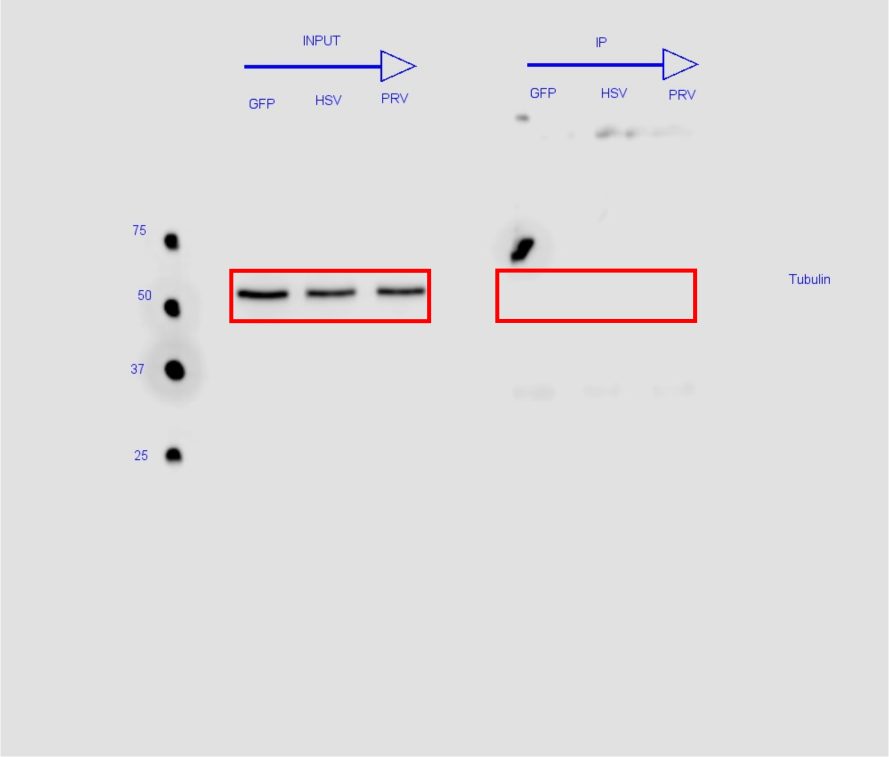

anti-Tubulin
